# *Drosophila* immune cells transport oxygen through PPO2 protein phase transition

**DOI:** 10.1038/s41586-024-07583-x

**Published:** 2024-06-26

**Authors:** Mingyu Shin, Eunji Chang, Daewon Lee, Nayun Kim, Bumsik Cho, Nuri Cha, Ferdinand Koranteng, Ji-Joon Song, Jiwon Shim

**Affiliations:** 1 Department of Life Science, College of Natural Science, Hanyang University, Seoul, Republic of Korea; 2 Department of Biological Sciences, KI for BioCentury, Korea Advanced Institute of Science and Technology (KAIST), Daejeon, Republic of Korea; 3 Research Institute for Natural Science, Hanyang University, Seoul, Republic of Korea; 4 Hanyang Institute of Bioscience and Biotechnology, Hanyang University, Seoul, Republic of Korea; 5 Hanyang Institute of Advanced BioConvergence, Hanyang University, Seoul, Republic of Korea

**Keywords:** Respiration, Haematopoiesis, Cellular imaging, Evolutionary developmental biology

## Abstract

Insect respiration has long been thought to be solely dependent on an elaborate tracheal system without assistance from the circulatory system or immune cells^1,2^. Here we describe that *Drosophila* crystal cells—myeloid-like immune cells called haemocytes—control respiration by oxygenating Prophenoloxidase 2 (PPO2) proteins. Crystal cells direct the movement of haemocytes between the trachea of the larval body wall and the circulation to collect oxygen. Aided by copper and a neutral pH, oxygen is trapped in the crystalline structures of PPO2 in crystal cells. Conversely, PPO2 crystals can be dissolved when carbonic anhydrase lowers the intracellular pH and then reassembled into crystals in cellulo by adhering to the trachea. Physiologically, larvae lacking crystal cells or PPO2, or those expressing a copper-binding mutant of PPO2, display hypoxic responses under normoxic conditions and are susceptible to hypoxia. These hypoxic phenotypes can be rescued by hyperoxia, expression of arthropod haemocyanin or prevention of larval burrowing activity to expose their respiratory organs. Thus, we propose that insect immune cells collaborate with the tracheal system to reserve and transport oxygen through the phase transition of PPO2 crystals, facilitating internal oxygen homeostasis in a process that is comparable to vertebrate respiration.

## Abstract (web summary)

*Drosophila* haemocytes collaborate with the tracheal system to reserve and transport oxygen through the phase transition of PPO2 crystals, facilitating internal oxygen homeostasis in a process that is comparable to vertebrate respiration.

## Main

Oxygen is an essential molecule for life^3^. The ability to transport oxygen has been a key driver of animal evolution. Accordingly, many oxygen-binding proteins and mechanisms for efficient gas exchange have evolved in the animal kingdom. In most vertebrates, oxygen is primarily bound to haemoglobin and carried in red blood cells, which circulate through the closed circulatory system, holding or releasing oxygen through the Bohr effect and differential partial pressures of oxygen^4,5^. Some invertebrates, such as mollusks and a few arthropod subphyla, possess haemocyanin, a type of oxygen carrier protein that freely circulates within the haemolymph for convective oxygen delivery without the assistance of any specific immune cell types^6,7^. In insects, it had been thought that a densely coordinated tracheal system was sufficient for gas exchange, and that immune cells specialized for respiration, or respiratory proteins, were unnecessary^2,8^.

In *Drosophila melanogaster*, immune cells called haemocytes are reminiscent of myeloid-lineage blood cells in vertebrates and are sentinels of innate immunity and stress responses^9^. There are three distinct haemocyte populations in *Drosophila* larvae: plasmatocytes, crystal cells and lamellocytes^10^. The majority (around 95%) of haemocytes are plasmatocytes, which are most analogous to vertebrate macrophages^11,12^. Crystal cells, named for their crystalline inclusions, account for the remaining 5% of haemocytes and have a critical role in melanization and wound healing; lamellocytes appear only during immune activation^13–15^. Larval haemocytes are derived from two evolutionarily conserved cell lineages: (1) the embryonic lineage of haemocytes that circulate in the larval haemolymph or proliferate and differentiate in haematopoietic pockets of the larval body wall; or (2) the larval lymph gland lineage through myeloid-like progenitors^9,16,17^.

Recent studies have shown that low levels of respiratory gases, including oxygen and carbon dioxide, induce the differentiation of a specific immune cell type in *Drosophila*^18,19^. However, whether this is related to the control of respiration by insect immune cells has been a critical open question in the field. Here we describe a cellular pathway involving a direct role for crystal cells in oxygen transport and acquisition through the phase separation and conversion of PPO2 proteins in response to oxygen variability to ensure animal growth and survival.

## Systemic hypoxia in larvae lacking crystal cells

**Fig. 1:**
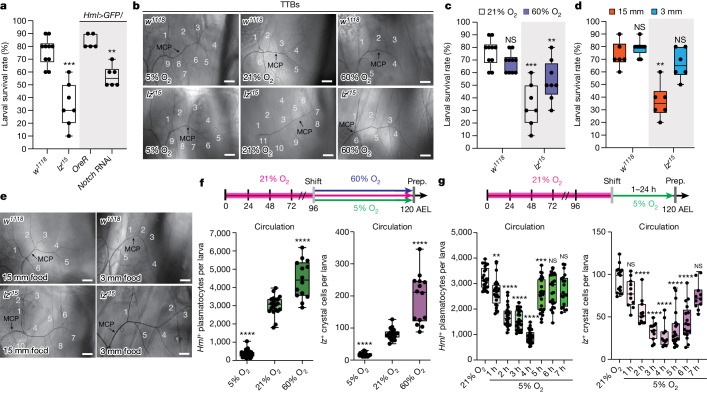
Crystal cells control internal oxygen homeostasis, and ambient oxygen determines haemocyte localization. **a**–**d**, Crystal cells are required for larval survival. **a**, The larval survival rates of control larvae or mutant larvae lacking crystal cells in normoxia (21% O_2_). **b**, Loss of crystal cells in *lz*^*r15*^ mutants resulted in increased tracheal TTBs, which was rescued by hyperoxia (60% O_2_). MCP, main cellular process. **c**, Attenuated larval survival rates of *lz*^*r15*^ mutants recovered under hyperoxic conditions. **d**, Reduced larval survival rates of *lz*^*r15*^ mutant in 15-mm-depth food (orange) were rescued by 3-mm-depth food (blue). **e**, The increase in TTBs of *lz*^*r15*^ mutant was rescued by 3-mm-depth food. **f**, Oxygen concentrations modulate the number of haemocytes in the haematopoietic pocket (sessile) or in the haemolymph (circulation). Top, schematics of the oxygen control experiments. Bottom, quantification of circulating *Hml*^+^ plasmatocytes (left) or *lz*^+^ crystal cells (right) shown in Extended Data Fig. 1e. Prep., preparation. **g**, Haemocytes translocate between the haematopoietic pocket (sessile) and the haemolymph (circulation) during acute hypoxia. Top, schematics of hypoxia experiments. Bottom, quantification of circulating *Hml*^+^ plasmatocytes (left) or *lz*^+^ crystal cells (right) shown in Extended Data Fig. 1m. Scale bars, 50 µm (**b** and **e**). Statistical analysis was performed using Mann–Whitney *U*-tests (**a**, **c**, **d** and **f**) and one-way analysis of variance (ANOVA) followed by Tukey’s post hoc analysis (**g**); NS, not significant; **P < 0.01, ***P < 0.001, ****P < 0.0001. Quantification of TTB numbers in **b** and **e** is shown in Supplementary Tables 1 and 2. The box and whisker plots show the median (centre line), 25% and 75% (box limits), and the maximum and minimum values (whiskers). Detailed genotypes, sample sizes, statistics and *Gal4* drivers are shown in Supplementary Table 5. Source Data

**Extended Data Fig. 1:**
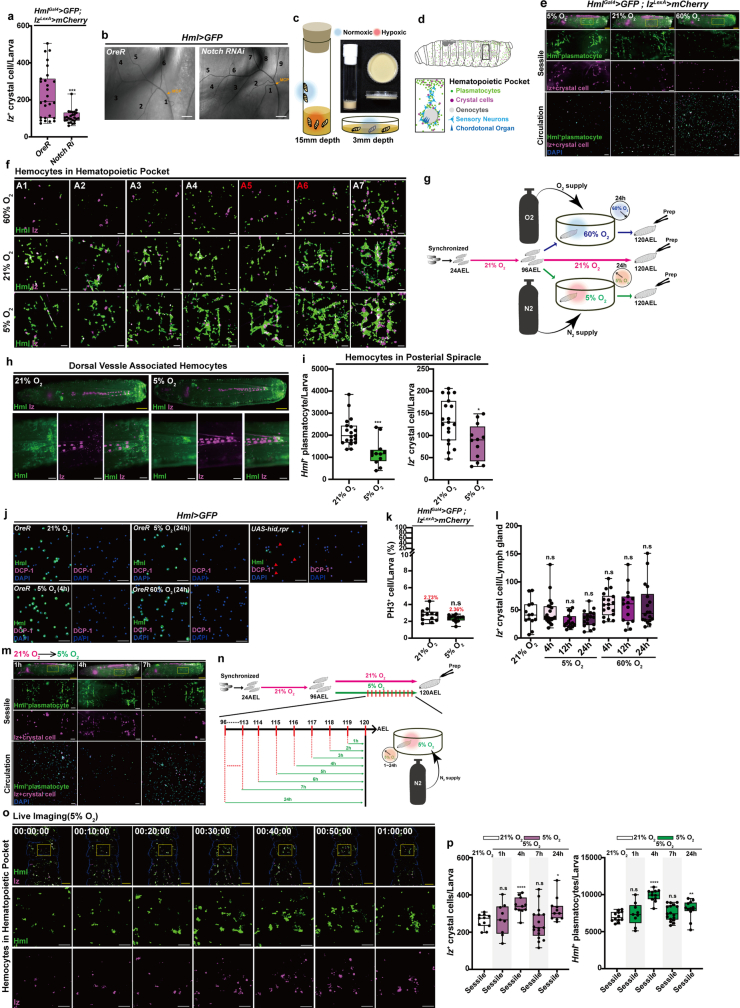
Hemocyte responses during 24-h hypoxia in the hematopoietic pocket. **a**–**b**. Specific depletion of crystal cells by *Notch* RNAi. (**a**) Expression of *Notch* RNAi driven by *Hml*^*Δ*^*-Gal4* specifically inhibited crystal cell development. (**b**) This genetic manipulation increased the number of TTBs. Quantification of TTB numbers is presented in Supplementary Tables 1 and 2. (**c**) Graphical illustration and image of food at a 15-mm depth in a plastic vial and food at a 3-mm depth in a 60-mm Petri dish. (**d**) Schematics of larval hemocyte distribution (top) and organization of the hematopoietic pocket (bottom). Lateral view of segment A6 is magnified to highlight components of the hematopoietic pocket (plasmatocytes, green; crystal cells, magenta; oenocytes, grey; sensory neurons, light blue; chordotonal organ, blue). Reproduced with permission from ref. ^10^, Elsevier. (**e**) Expression patterns of hemocytes (*Hml*^*+*^ plasmatocytes, green; *lz*^*+*^ crystal cells, magenta) (*Hml*^*Δ*^*-Gal4 UAS-EGFP; lz-LexA LexAop-mCherry*) in whole larvae (top), sessile hemocytes in the hematopoietic pocket (middle), or circulating hemocytes upon bleeding (bottom). (**f**) Hemocytes adhered to the hematopoietic pocket during hypoxia. Larvae carrying *Hml*^*Δ*^*-Gal4 UAS-EGFP; Lz-LexA LexAop-mCherry* were synchronized and transferred to the desired oxygen conditions (21%, 5%, or 60% O_2_) at 96 h after egg laying (AEL), and phenotypes were observed at 120-h AEL. The numbers and localization patterns of hemocytes (*Hml*^+^ plasmatocytes, green; *lz*^+^ crystal cells, magenta) in the hematopoietic pocket (A1–A7) exposed to 60% (top), 21% (middle), or 5% (bottom) O_2_. The A5 or A6 segment (red) recruited the largest number of hemocytes. (**g**) Schematic of oxygen control experiments shown in Fig. 1f. (**h**) The numbers and localization patterns of hemocytes (*Hml*^+^ plasmatocytes, green; *lz*^+^ crystal cells, magenta) in the posterior dorsal vessels exposed to 21% (left) or 5% (right) O_2_ for 24 h in a whole larva (top) or dorsal vessel-associated cluster (bottom). (**i**) Quantification of hemocytes (*Hml*^+^ plasmatocytes, left; *lz*^+^ crystal cells, right) in the posterior spiracles exposed to 21% or 5% O_2_. *Hml*^+^ plasmatocytes (left) and *lz*^+^ crystal cells (right) were counted separately. White bars indicate 21% O_2_, and green or magenta bars indicate 5% O_2_. (**j**) Hemocytes do not undergo apoptosis during hypoxia or hyperoxia. Expression of an apoptosis marker (anti-DCP-1) in hemocytes exposed to 21%, 5%, or 60% O_2_ for 24 h from 96-h AEL to 120-h AEL (*Hml*^*Δ*^*-Gal4 UAS-EGFP*) (*Hml*^+^ plasmatocytes, green; DCP-1, magenta; DAPI, blue). Hemocytes expressing the pro-apoptotic genes *hid* and *rpr* (*Hml*^*Δ*^*-Gal4 UAS-EGFP UAS-Hid, Rpr*) are positive controls. Arrowheads (red) indicate DCP-1-positive hemocytes. (**k**) The number of hemocytes expressing a mitosis marker, anti-phospho-histone H3 (PH3) did not change during hypoxia (5% O_2_). (**l**) The number of crystal cells in the lymph gland was not altered in hypoxia or hyperoxia (60% O_2_). Quantification of lymph gland crystal cells exposed to 21%, 5% or 60% O_2_ for the indicated lengths of time. (**m**) Expression patterns of hemocytes (*Hml*^+^ plasmatocytes, green; *lz*^+^ crystal cells, magenta) (*Hml*^*Δ*^*-Gal4 UAS-EGFP; lz-LexA LexAop-mCherry*) in whole larvae (top), sessile hemocytes in the hematopoietic pocket (middle), and circulating hemocytes upon bleeding (bottom). (**n**) Schematic of oxygen control experiments shown in Fig. 1g. (**o**) Confocal live imaging of sessile hemocytes (*Hml*^*Δ*^*-Gal4 UAS-EGFP; lz-LexA LexAop-mCherry*) during 1-h hypoxia. Images were captured every 10 min up to 1 h. Whole larvae (top), *Hml*^+^ plasmatocytes (middle), and *lz*^+^ crystal cells (bottom). Magnified images of yellow inset (top) are indicated below (middle and bottom). (**p**) The number of sessile hemocytes obtained by the scraping assay. Compared to controls at normoxia (white), the numbers of sessile hemocytes increased at 4- and 24-hour hypoxia (crystal cell, magenta; plasmatocyte, green). Scale bars: yellow, 500 µm; white, 50 µm. n.s. (not significant), *P < 0.05, **P < 0.01, ***P < 0.001, ****P < 0.0001. Extended Data Fig. 1a, i, k, l, and p were analysed using the Mann–Whitney test. Box and whiskers plots in **1a**, **1i**, **1k**, **1l**, and **1p** denote maximum, 25%, median, 75%, and minimum values, respectively. Schematic diagrams in Extended Data Fig. 1c, g, and n were created with BioRender.com. Detailed genotypes, sample sizes, and *Gal4* drivers with corresponding target tissues are listed in Supplementary Table 5. Source Data

*lozenge*^*r15*^ (*lz*^*r15*^) *Drosophila* mutants lacking crystal cells, one of the three types of haemocytes^20–22^, were unhealthy and challenging to maintain compared with the wild-type (WT) flies. While 76% of WT larvae successfully progressed through the larval stages, only 34% of *lz*^*r15*^ mutants survived to the third instar under normal laboratory conditions at 25 °C (Fig. 1a). This phenotype was also observed in blood-cell-specific *Notch* inhibition, in which crystal cell differentiation was specifically abolished^19^ (Fig. 1a and Extended Data Fig. 1a). Morphologically, *lz*^*r15*^ mutants or larvae expressing *Notch* RNAi displayed an increased number of thick terminal branches (TTBs) in the trachea under normoxic conditions (21% O_2_) (Fig. 1b, Extended Data Fig. 1b and Supplementary Tables 1 and 2). As TTB growth is influenced by internal oxygen levels^23,24^, we investigated whether the increased TTB development in *lz*^*r15*^ mutants was associated with oxygen availability. Consistent with previous reports^23^, hypoxia (5% O_2_) significantly increased the number of TTBs to numbers comparable with those observed in *lz*^*r15*^ mutants (Fig. 1b and Supplementary Tables 1 and 2). Culturing *lz*^*r15*^ mutants in hypoxia did not further increase the number of TTBs (Fig. 1b and Supplementary Tables 1 and 2). However, hyperoxia (60% O_2_) completely restored TTB numbers in *lz*^*r15*^ mutant larvae to levels similar to WT larvae in normoxia (Fig. 1b and Supplementary Tables 1 and 2). Furthermore, the reduced larval survival rate of *lz*^*r15*^ mutants was reversed by hyperoxia (Fig. 1c). These results suggest that *lz*^*r15*^ mutant larvae experience systemic hypoxia due to the absence of crystal cells.

*Drosophila* larvae spend a substantial portion of their developmental stage burrowing for food, which potentially exposes them to hypoxic stress. Considering the reduced larval survival rate and increased TTBs observed in *lz*^*r15*^ mutants, we hypothesized that burrowing behaviour and limited oxygen availability during feeding contributed to the phenotypes of *lz*^*r15*^ mutants. To alleviate the inherent hypoxia, we introduced a shallow food source (3 mm depth) in a 60 mm dish containing the same amount of food as provided in regular plastic vials (Methods and Extended Data Fig. 1c). Notably, the survival rate of *lz*^*r15*^ mutant larvae cultured on the shallow food source was comparable to that of WT animals (*w*^*1118*^) raised in plastic vials (food depth, 15 mm) (Fig. 1d). Furthermore, the 3-mm-depth food rescued the increased TTB phenotype observed in *lz*^*r15*^ mutants (Fig. 1e and Supplementary Tables 1 and 2). These results indicate that shallow food alleviated the characteristic hypoxic phenotypes of *lz*^*r15*^ mutants, enabling them to achieve fitness levels similar to those of WT larvae. Importantly, these findings suggest that crystal cells have a crucial role in maintaining internal oxygen levels during burrowing behaviour associated with feeding during development.

## Ambient O_2_ shapes the haemocyte niche

Based on the apparent significance of crystal cells in internal oxygen level maintenance, we attempted to observe the most striking phenotypes of haemocytes at varying oxygen concentrations to identify the mechanisms underlying crystal-cell-mediated oxygen homeostasis.

During larval stages, two types of haemocytes—circulating haemocytes in the haemolymph and sessile haemocytes colonizing resident tissues—collectively maintain their numbers (Extended Data Fig. 1d). Despite their constant movement to and from resident tissues, the numbers of circulating or sessile haemocytes are maintained at constant levels unless the immune system is challenged^25–27^. Under normoxic conditions, at 120 h after egg laying (AEL), third-instar larvae displayed consistent numbers of circulating haemocytes when visualized using markers specific to plasmatocytes (*Hml*^+^) or crystal cells (*lz*^+^) (Fig. 1f and Extended Data Fig. 1e–g). However, when larvae were cultured under hypoxic conditions, most haemocytes were withdrawn from circulation, instead accumulating in the haematopoietic pocket, which comprises segmentally repeated microenvironments for haemocyte adherence in the larval body wall (Fig. 1f and Extended Data Fig. 1e–g). Conversely, hyperoxia markedly increased the number of circulating haemocytes, including plasmatocytes and crystal cells, while reducing sessile haemocytes at the haematopoietic pocket (Fig. 1f and Extended Data Fig. 1e–g). Haemocyte clusters in the posterior dorsal vessels or posterior spiracles, which serve as additional sites for haemocyte adherence^27^, remained unaffected or declined, respectively, in response to hypoxia (Extended Data Fig. 1h,i). The observed changes in haemocyte behaviour were not associated with cell death, proliferation or alterations in larval haematopoiesis in the lymph gland or circulation under hypoxic or hyperoxic conditions (Extended Data Fig. 1j–l), ruling out other contributors to the observed haemocyte responses to changes in oxygen levels.

**Extended Data Fig. 2:**
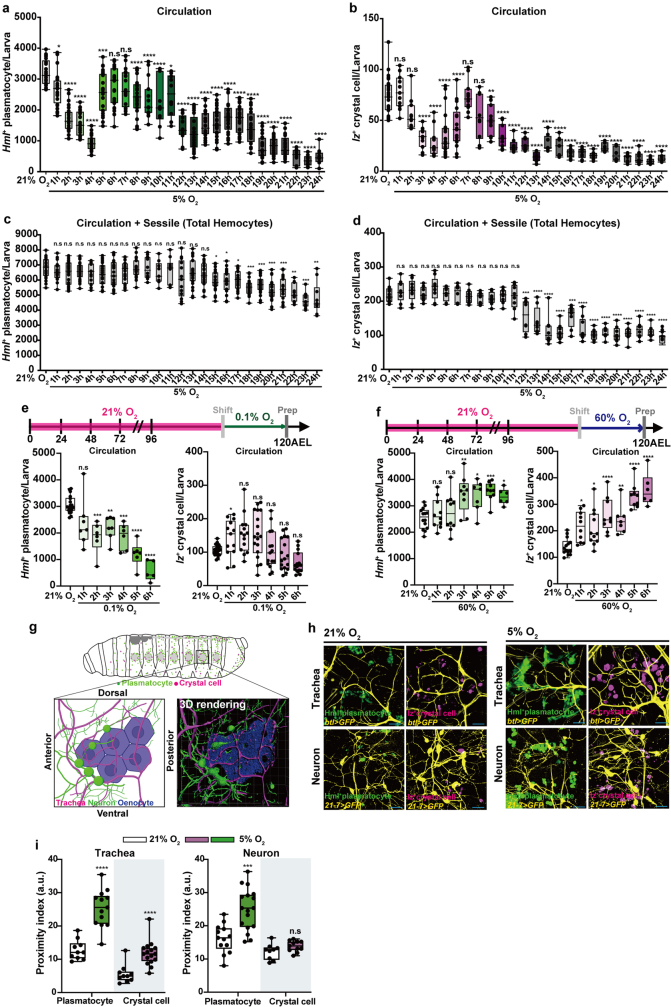
Hemocyte responses until 24-h hypoxia or under various oxygen concentrations in the circulation. **a**–**b**. Larvae were synchronized and reared in 21% O_2_ until moved hourly to 5% O_2_ over 1 to 24 h before dissection and observation at 120-h AEL. Quantification of (**a**) circulating *Hml*^+^ plasmatocytes (left) or (**b**) *lz*^+^crystal cells (right) in each oxygen condition. **c**–**d**. Hematopoiesis is not altered in 5% O_2_ up to 12 h. Total hemocyte numbers of larvae carrying *Hml*^*Δ*^*-Gal4 UAS-EGFP; lz-LexA LexAop-mCherry* exposed to 5% O_2_ for the indicated lengths of time up to 24 h. Quantification of total plasmatocytes (*Hml*^+^) (**c**) or crystal cells (*lz*^+^) (**d**) grown in each condition. **e**–**f**. Oxygen levels determine hemocyte dynamics between the hematopoietic pocket (sessile) and the haemolymph (circulation). Synchronized larvae carrying *Hml*^*Δ*^*-Gal4 UAS-EGFP; lz-LexA LexAop-mCherry* were reared in 21% O_2_ and shifted to 0.1% (anoxia) or 60% (hyperoxia) O_2_ for the indicated amounts of time, and the phenotypes were observed at 120-h AEL. (**e**) Schematics of anoxia experiments; a vertical grey bar indicates the time of the shift between oxygen conditions (top). Quantitation of circulating plasmatocytes (*Hml*^+^) (left) or crystal cells (*lz*^+^) (right) exposed to 0.1% O_2_ for the indicated numbers of hours. (**f**) Schematics of hyperoxia experiments; a vertical grey bar indicates the time of the shift between oxygen conditions (top). Quantification of circulating plasmatocytes (*Hml*^+^) (left) or crystal cells (*lz*^+^) (right) exposed to 60% O_2_ for the indicated hours. (**g**) Lateral view of segment A6 is magnified to highlight components of the hematopoietic pocket (neuron, green; trachea, magenta; oenocyte, blue). Schematic or confocal images of segment A6 labelled with *btl-LexA LexAop-mCherry; 21-7-Gal4 UAS-mCD8::GFP* (bottom). Reproduced with permission from ref. ^10^, Elsevier. **h**–**i**. Increased numbers of hemocytes were closely associated with PNS neurons or the trachea during hypoxia. (**h**) The proximity of hemocytes to tissues in the hematopoietic pocket marked by trachea-*Hml*^+^ plasmatocytes (*btl-Gal4 UAS-GFP; Hml*^*Δ*^*-dsRed*), trachea-*lz*^+^crystal cells (*btl-Gal4 UAS-GFP; lz-LexA LexAop-mCherry*), PNS neuron-*Hml*^+^ plasmatocytes (*Hml*^*Δ*^*-LexA LexAop-mCherry; 21-7-Gal4 UAS-mCD8::GFP*), or PNS neuron-*lz*^+^ crystal cells (*21-7-Gal4 UAS-mCD8::GFP; lz-LexA LexAop-mCherry*) in normoxia or 4-h hypoxia. (**i**) Quantification of the proximity index (a.u.) of the trachea and hemocytes (left) or PNS neurons and hemocytes (right). Scale bars: blue, 20 µm. n.s. (not significant), *P < 0.05, **P < 0.01, ***P < 0.001, ****P < 0.0001. Extended Data Fig. 2a–f were analysed by one-way ANOVA followed by Tukey’s post hoc test. Box and whiskers plots in **2a**–**f** and **2i** denote maximum, 25%, median, 75%, and minimum values, respectively. Source Data

To investigate haemocyte dynamics in detail, we performed a synchronization experiment with larvae under normoxic conditions at 25 °C, followed by exposure to hypoxia for different durations (1–24 h) before dissection and observation at 120 h AEL. For example, in the 4 h hypoxia group, larvae were synchronized and cultured in normoxia until 116 h AEL, when they were transferred to 5% O_2_ until 120 h AEL. By measuring the number of circulating plasmatocytes and crystal cells at each hour of hypoxia exposure up to 24 h (Methods), we observed a gradual decrease in the number of circulating plasmatocytes and crystal cells starting at 1 h of hypoxia (Fig. 1g and Extended Data Fig. 1m,n). Live imaging of larval haemocytes validated that both plasmatocytes and crystal cells progressively accumulated in the haematopoietic pocket within the first hour of hypoxia exposure (Extended Data Fig. 1o). This gradual decrease in circulating plasmatocytes and crystal cells continued until the 4 h timepoint, when the lowest numbers of the circulating population and the highest numbers of sessile haemocytes were observed (Fig. 1g and Extended Data Figs. 1m,p). Notably, the number of circulating plasmatocytes and crystal cells naturally recovered to normal levels after 5 to 7 h of hypoxia, followed by subsequent oscillations, creating a damped oscillatory-like pattern over the 24 h period (Extended Data Fig. 2a,b). The total numbers of plasmatocytes or crystal cells did not change during the first 12 h of hypoxia (Extended Data Fig. 2c,d), indicating that haemocyte development was not hindered within this timeframe. Thus, our analysis focused on the effects observed between 1 and 7 h of hypoxia (Fig. 1g and Extended Data Fig. 1m,n), when haemocytes translocate at least once.

Under anoxic conditions (0.1% O_2_), plasmatocytes gradually disappeared from circulation and did not reappear until after 6 h of exposure. The adherence of crystal cells to the haematopoietic pocket observed at 5% O_2_ was also lost (Extended Data Fig. 2e). Conversely, hyperoxia maintained a higher number of circulating haemocytes compared with normoxic conditions (Extended Data Fig. 2f). Together, these findings demonstrate that haemocyte dynamics are modified by environmental oxygen levels, leading to coordinated positional changes between the haematopoietic pocket and circulation.

## Trachea drives haemocyte translocation

**Extended Data Fig. 3:**
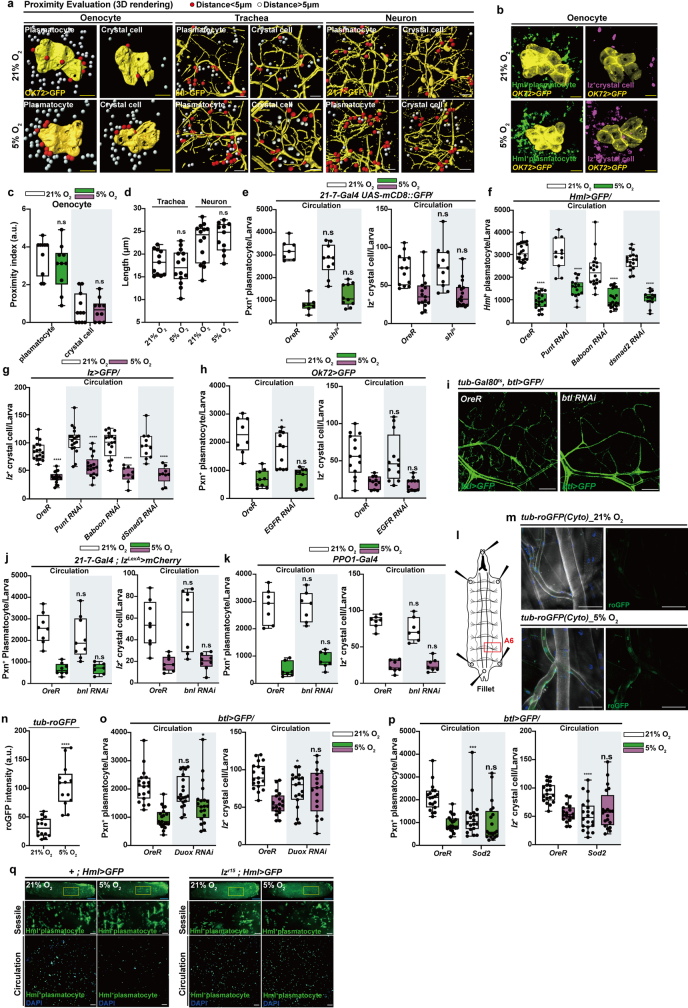
Trachea-induced reactive oxygen species activate hemocyte adherence to the hematopoietic pocket. (**a**) Hemocytes increased their association with the trachea (*btl*^+^) or PNS neurons (*21-7*^+^) in 5% O_2_ (bottom) compared with 21% O_2_ (top) but not with oenocytes (*OK72*^+^). Proximity evaluation in 3D images between oenocytes (*OK72*^+^ oenocyte, yellow) and hemocytes (white or red) (left), the trachea (*btl*^+^ trachea, yellow) and hemocytes (white or red) (middle), or PNS neurons (*21-7*^+^ neuron, yellow) and hemocytes (white or red) (right). Red dots indicate hemocytes within a 5-µm radius of each component in the hematopoietic pocket and are quantified in Fig. 2c or Extended Data Fig. 3c. **b**–**c**. The number of hemocytes adhering to oenocytes remained constant. (**b**) Proximity of hemocytes and oenocytes labelled with oenocyte–plasmatocyte markers (*OK72-Gal4 UAS-mCD8::GFP; Hml*^*Δ*^*-dsRed*) or oenocyte–crystal cell markers (*OK72-Gal4 UAS-mCD8::GFP; lz-LexA LexAop-mCherry*) at 21% O_2_ or 5% O_2_ for 4 h. (**c**) Quantification of proximity index (a.u) between oenocytes and hemocytes. (**d**) The total length of the trachea or PNS neurons remained unchanged at 5% O_2_. (**e**) Silencing PNS neurons did not alter hemocyte movement upon hypoxia. The number of circulating hemocytes in controls (*21-7-Gal4* UAS-*mCD8::GFP*) or after silencing PNS neurons (*21-7-Gal4 UAS-mCD8::GFP UAS-shi*^*ts*^). **f**–**g**. The Activin-β (Actβ)/dSmad2 pathway in PNS neurons did not change hemocyte localization at 5% O_2_. (**f**) Quantification of circulating plasmatocytes (*Hml*^+^) in plasmatocyte-specific downregulation of three genes: *punt, baboon*, and *dSmad2* (*Hml*^*Δ*^*-Gal4 UAS-EGFP, UAS-put RNAi or UAS-babo RNAi, UAS-dSmad2 RNAi*). (**g**) Quantification of circulating crystal cells (*lz*^+^) in crystal-cell-specific downregulation of three genes: *punt, baboon*, and *dSmad2* (*lz-Gal4 UAS-EGFP, UAS-put RNAi, UAS-babo RNAi, UAS-dSmad2 RNAi*). (**h**) The decreased development of oenocytes did not influence crystal cell or plasmatocyte localization at 5% O_2_. Quantification of circulating Pxn^+^ plasmatocytes (left) or lz^+^ crystal cells (right) in each condition. (**i**) Silencing of the FGF receptor, *btl*, decreased tracheal branching. Distribution of the trachea (*btl*^+^ trachea, green) in controls (*tub-Gal80*^*ts*^*; btl-Gal4 UAS-GFP*) or *btl* RNAi in the trachea (*tub-Gal80*^*ts*^*; btl-Gal4 UAS-GFP, UAS-btl RNAi*). **j**–**k**. The FGF ligand, *bnl*, was unnecessary for hemocyte movement during hypoxia. (**j**) Expression of RNAi against *bnl* in peripheral neurons (*21-7-Gal4; lz-LexA LexAop-mCherry*) or (**k**) in crystal cells (*PPO1-Gal4*) did not alter the attachment of hemocytes during 4-h hypoxia. **l**-**n**. Reactive oxygen species (ROS) levels were induced after 4-h hypoxia. (**l**) A schematic diagram of fillet dissection. The A6 region is marked in red. (**m**) Expression of *roGFP* (green) in the trachea exposed to 21% O_2_ (top) or 5% O_2_ (bottom) for 4 h. (**n**) Quantification of *roGFP* in **(m**). **o**–**p**. Scavenging ROS altered hemocyte translocation to the hematopoietic pocket during hypoxia. (**o**) Expression of RNAi against *Duox* suppressed the localization of both Pxn^+^ plasmatocytes (left) and *lz*^+^ crystal cells (right) to the hematopoietic pocket after 4-h hypoxia (*btl-Gal4 UAS-GFP UAS-Duox RNAi; lz-LexA LexAop-mCherry*). (**p**) Expression of *superoxide dismutase 2* (*Sod2*) inhibited the localization of both Pxn^+^ plasmatocytes (left) and *lz*^+^ crystal cells (right) to the hematopoietic pocket following 4-h hypoxia (*btl-Gal4 UAS-GFP UAS-Sod2; lz-LexA LexAop-mCherry*). (**q**) Expression patterns of *Hml*^+^ plasmatocytes in controls (*Hml*^*Δ*^*-Gal4 UAS-EGFP*) (left) or *lz*^*r15*^ mutants (*lz*^*r15*^*; Hml*^*Δ*^*-Gal4 UAS-EGFP*) (right) in whole larvae (top), *Hml*^+^ sessile hemocytes in the hematopoietic pocket (middle), or *Hml*^+^ circulating hemocytes upon bleeding (bottom), corresponding to Fig. 2c. White bars indicate 21% O_2_, green (plasmatocytes) or magenta (crystal cells) bars indicate 5% O_2_. Scale bars: yellow, 20 µm; white, 50 µm; blue, 500 µm. n.s (not significant), *P < 0.05, **P < 0.01, ***P < 0.001, ****P < 0.0001. Extended Data Fig. 3c, d, f, g, and n were analysed using the Mann–Whitney test. Extended Data Fig. 3e, h, j, k, o, and p were analysed by two-way ANOVA followed by Bonferroni’s post hoc test. Box and whiskers plots in **3c**–**h**, **3j**–**k**, and **3n**–**p** denote maximum, 25%, median, 75%, and minimum values, respectively. Source Data

The haematopoietic pocket^26,27^ represents a complex microenvironment in which multiple cell types, including those of the peripheral nervous system (PNS), oenocytes (a group of hepatocyte-like cells in *Drosophila* larvae) and the trachea, interact (Extended Data Fig. 2g). To determine the specific cell type to which haemocytes adhere during changes in ambient oxygen levels, we examined the number of plasmatocytes (*Hml*^+^) or crystal cells (*lz*^+^) within a 5 μm radius of three representative tissues in the haematopoietic pocket after 4 h of hypoxia, when the highest number of haemocytes was observed in the haematopoietic pocket (Extended Data Fig. 1p). Under hypoxic conditions, both plasmatocytes and crystal cells displayed an increased association with the trachea or PNS neurons (Extended Data Figs. 2h,i and 3a), whereas the proximity of haemocytes and oenocytes remained unaltered (Extended Data Fig. 3b,c). There were no significant alterations in the length of the trachea or neurons after 4 h of hypoxia (Extended Data Fig. 3d), indicating that the enhanced proximity of haemocytes to the trachea or PNS neurons observed during hypoxia is not attributable to structural changes in the haematopoietic pocket.

**Fig. 2:**
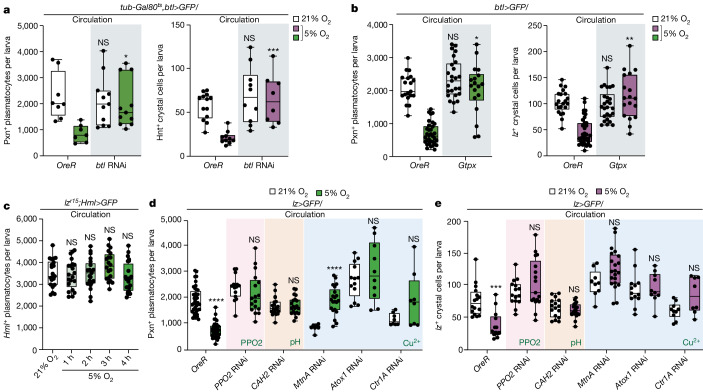
The trachea and crystal cells are required for haemocyte adherence during hypoxia. **a**, Inhibition of tracheal development suppressed haemocyte movement during hypoxia (5% O_2_). Quantification of the number of circulating haemocytes (Pxn^*+*^ plasmatocytes, green; *lz*^*+*^ crystal cells, magenta) in controls or after depletion of tracheal branches by knockdown of the FGF receptor *btl* in the trachea. **b**, H_2_O_2_ in the trachea was essential for haemocyte movement in hypoxia. Quantification of the number of circulating haemocytes (Pxn^*+*^ plasmatocytes, green; *lz*^*+*^ crystal cells, magenta) by expression of *Gtpx* in the trachea. **c**, Larvae lacking crystal cells did not trigger plasmatocyte movement to the haematopoietic pocket after hypoxia. Quantification of circulating *Hml*^+^ plasmatocytes in *lz*^*r15*^ mutants under normoxic or hypoxic conditions. **d**,**e**, *PPO2*, *CAH2* and copper regulators were critical for crystal cell movement. Crystal-cell-specific downregulation of *PPO2*, *CAH2*, *MtnA*, *Atox1* or *Ctr1A* inhibited haemocyte movement at 4 h of hypoxia. Quantification of circulating Pxn^+^ plasmatocytes (**d**) or lz^+^ crystal cells (**e**) in each condition and genetic background. White bars, normoxia (21% O_2_); green or magenta bars, hypoxia (5% O_2_). *P < 0.05. Statistical analysis was performed using two-way ANOVA followed by Bonferroni’s post hoc test (**a** and **b**), one-way ANOVA followed by Tukey’s post hoc test (**c**) and Mann–Whitney *U*-tests (**d** and **e**). The box and whisker plots show the median (centre line), 25% and 75% (box limits), and the maximum and minimum values (whiskers). Detailed genotypes, sample sizes, statistics and *Gal4* drivers are provided in Supplementary Table 5. Source Data

Sensory neurons of the PNS act as a trophic microenvironment for resident haemocytes by activating the Activin-β (Actβ)–dSmad2 pathway^26^. Inactivating PNS neurons or disrupting plasmatocyte- or crystal-cell-mediated TGF-β–SMAD signalling through the expression of interfering RNA (RNAi) against *dSmad2*, *baboon* (*babo*) or *punt* (*put*) did not alter the reduction in circulating plasmatocytes or crystal cells observed after 4 h of hypoxia (Extended Data Fig. 3e–g). Moreover, suppression of oenocyte cell fate by inhibiting the epidermal growth factor receptor (*EGFR*) did not affect haemocyte movement (Extended Data Fig. 3h). However, when tracheal branching was suppressed by the *breathless* (*btl*) FGF receptor RNAi in the trachea^28^ (Extended Data Fig. 3i), the number of circulating plasmatocytes and crystal cells remained comparable to that of the control group after 4 h of hypoxia (Fig. 2a). This phenotype was not mediated by the *branchless* (*bnl*) FGF ligand (Extended Data Fig. 3j,k). These results suggest that haemocytes do not relocate to the haematopoietic pocket when tracheal branching is disrupted. In the trachea, the levels of hydrogen peroxide (H_2_O_2_)—monitored using *roGFP*^29^, a fluorescent sensor for intracellular H_2_O_2_—were significantly induced after 4 h of hypoxia (Extended Data Fig. 3l–n). When H_2_O_2_ in the trachea was reduced by expression of the reactive oxygen species scavenger, *Gtpx*, or by an RNAi against *Dual oxidase* (*Duox*)^30^, haemocyte movement to the haematopoietic pocket was suppressed after 4 h hypoxia (Fig. 2b and Extended Data Fig. 3o). Moreover, overexpression of *Sod2*, which catalyses the conversion of superoxide radicals into H_2_O_2_, reduced the number of circulating haemocytes under normoxia but did not induce haemocyte translocation to the haematopoietic pocket during hypoxia (Extended Data Fig. 3p).

Taken together, these findings demonstrate that H_2_O_2_ produced in the trachea within the haematopoietic pocket induces interactions between haemocytes and the trachea, independent of developmental adhesion mediated by the PNS neurons.

## Crystal cells control haemocyte movement

**Extended Data Fig. 4:**
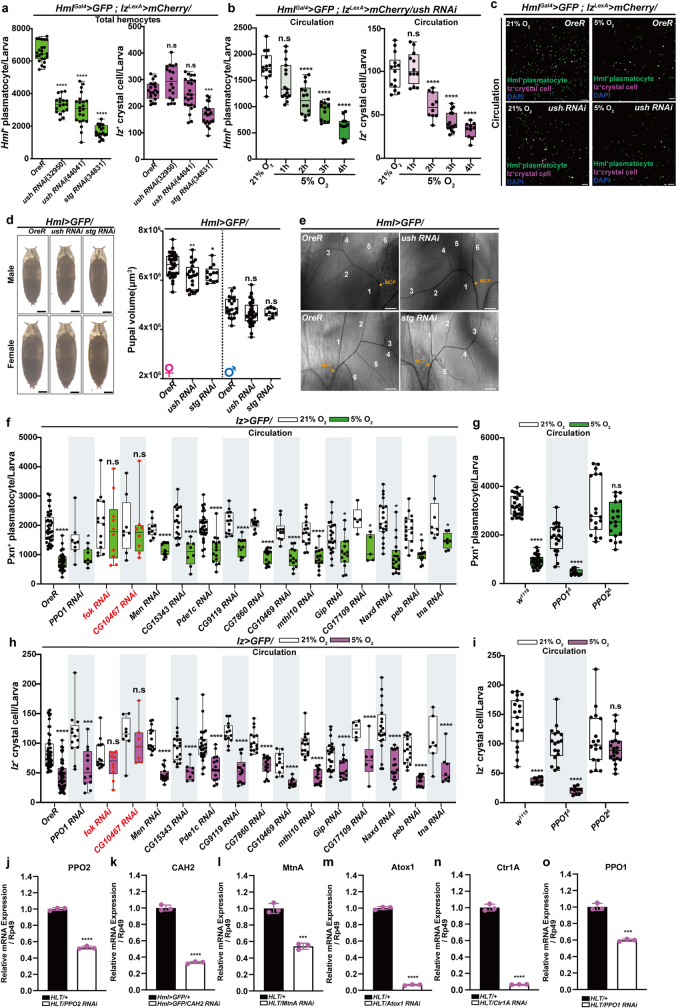
Identification of genes involved in crystal cell function. (**a**) Plasmatocyte-specific knockdown of *u-shaped* (*ush*) or *string* (*stg*) decreased plasmatocyte numbers. An RNAi-based screen targeted the following plasmatocyte proliferation genes: *ush* (*Hml*^*Δ*^*-Gal4 UAS-EGFP; lz-LexA LexAop-mCherry, UAS-ush RNAi* (BL32950, BL44041) or *UAS-stg RNAi* (BL34831)). Quantification of total *Hml*^+^ plasmatocytes (left) or *lz*^+^ crystal cells (right) in each genetic background. **b**–**c**. Decreased plasmatocyte numbers did not alter crystal cell localization at 5% O_2_. (**b**) Quantification of circulating *Hml*^+^ plasmatocytes (left) or *lz*^+^ crystal cells (right) in *Hml*^*Δ*^*-Gal4, UAS-ush RNAi* (BL44041). (**c**) The numbers and localization patterns of hemocytes (*Hml*^+^ plasmatocytes, green; *lz*^+^ crystal cells, magenta) in controls (*Hml*^*Δ*^*-Gal4 UAS-EGFP; lz-LexA LexAop-mCherry)* or in *ush* RNAi (*Hml*^*Δ*^*-Gal4, UAS-EGFP UAS-ush RNAi; lz-LexA LexAop-mCherry)* animals. **d**–**e**. Decreased plasmatocyte numbers did not mimic hypoxic animal phenotypes. (**d**) Expression of RNAi against *ush* (BL44041) or *stg* (BL34831) did not alter pupal volume. (**e**) These genetic backgrounds did not increase the TTB numbers. Quantification of TTB numbers is presented in Supplementary Tables 1 and 2. **f**–**i**. An RNAi-based screen targeted the top 20 crystal-cell-specific genes: *PPO1, fok, CG10467, Men, CG15343, Pde1c, CG9119, CG7860, CG10469, mthl10, Gip, CG17109, Naxd, peb, tna* (*lz-Gal4 UAS-EGFP, UAS-PPO1 RNAi, UAS-fok RNAi, UAS-CG10467 RNAi, UAS-Men RNAi, UAS-CG15343 RNAi, UAS-Pde1c RNAi, UAS-CG9119 RNAi, UAS-CG7860 RNAi, UAS-CG10469 RNAi, UAS-mthl10 RNAi, UAS-Gip RNAi, UAS-CG17109 RNAi, UAS-Naxd RNAi, UAS-peb RNAi*, and *UAS-tna RNAi*). Quantification of circulating Pxn^+^ plasmatocytes (**f**) or *lz*^+^ crystal cells (**h**) in each condition and genetic background. Red dotted bars indicate genes, including those of unknown function (*fok* and *CG10467*), that disrupted hemocyte movement to the hematopoietic pocket at 4 h in 5% O_2_. *PPO1*^*Δ*^ or *PPO2*^*Δ*^ mutants recapitulated the *PPO1* or *PPO2* RNAi phenotypes. Quantification of circulating Pxn^+^ plasmatocytes (**g**) or *lz*^+^ crystal cells (**i**) in each condition and genetic background. **j**–**o**. RNA interference (RNAi) efficiency of *Prophenoloxidase 2* (*PPO2, Hml*^*LineageTracing(LT)*^*-Gal4 UAS-PPO2 RNAi*) (**j**), *Carbonic anhydrase 2* (*CAH2*, *Hml*^*Δ*^*-Gal4 UAS-CAH2 RNAi*) (**k**), *Metallothionein A* (*MtnA, Hml*^*LT*^*-Gal4 UAS-MtnA RNAi*) (**l**), *Antioxidant 1 copper chaperone* (*Atox1, Hml*^*LT*^*-Gal4 UAS-Atox1 RNAi*) (**m**), *Copper transporter 1* *A* (*Ctr1A, Hml*^*LT*^*-Gal4 UAS-Ctr1A RNAi*) (**n**), or *Prophenoloxidase 1* (*PPO1, Hml*^*LT*^*-Gal4 UAS-PPO1 RNAi*) (**o**). White bars indicate 21% O_2_, and green or magenta bars indicate 5% O_2_. Scale bar: black, 500 µm; white, 50 µm. n.s (not significant), *P < 0.05, **P < 0.01, ***P < 0.001, ****P < 0.0001. Extended Data Fig. 4a, d, f, g, h, and i were analysed using the Mann–Whitney test. Extended Data Fig. 4b was analysed by one-way ANOVA followed by Tukey’s post hoc test. Extended Data Fig. 4j–o were analysed by Student’s *t* test. Box and whiskers plots in **4a**, **4b**, **4d**, and **4f**–**i** denote maximum, 25%, median, 75%, and minimum values, respectively. Error bars in **4j**–**o** represent standard deviations (SDs). Source Data

Given the hypoxic phenotypes observed in *lz*^*r15*^ mutants lacking crystal cells, we investigated whether crystal cells have a role in mobilizing haemocytes in collaboration with the trachea in response to varying oxygen availability. Notably, *lz*^*r15*^ mutants did not trigger the translocation of plasmatocytes to the haematopoietic pocket after 4 h of hypoxia, and the number of circulating plasmatocytes remained unchanged compared with the levels under normoxic conditions (Fig. 2c and Extended Data Fig. 3q). Conversely, when plasmatocytes were reduced by the loss of *u-shaped* (*ush*) or *string* (*stg*), which selectively suppresses the number of plasmatocytes^31–34^ (Extended Data Fig. 4a), the remaining plasmatocytes and crystal cells still disappeared from circulation after 4 h of hypoxia (Extended Data Fig. 4b,c). Furthermore, the reduced number of plasmatocytes did not alter animal growth or TTB development (Extended Data Fig. 4d,e). These results suggest that crystal cells, rather than plasmatocytes, are critical for the movement of themselves and plasmatocytes to the haematopoietic pocket and for internal oxygen homeostasis.

To gain insights into the molecular mechanisms underlying the crystal-cell-dependent movement of haemocytes to the haematopoietic pocket, we focused on analysing mature crystal cell marker genes from single-cell RNA-sequencing data^34,35^ (Supplementary Table 3). Among the 20 crystal-cell-enriched genes identified, the crystal-cell-specific downregulation of seven genes hindered the relocation of both plasmatocytes and crystal cells to the haematopoietic pocket after 4 h of hypoxia (Fig. 2d,e, Extended Data Fig. 4f–o and Supplementary Table 4). These downregulated genes included *PPO2*, which encodes an innate immune protein catalysing the melanization process, *Metallothionein A* (*MtnA*), *fledgling of Kpl38B* (*fok*), *CG10467*, *Antioxidant 1 copper chaperone* (*Atox1*), *Copper transporter 1A* (*Ctr1A*) and *Carbonic anhydrase 2* (*CAH2*), which encodes an enzyme converting carbon dioxide to bicarbonate and protons. Knockdown of *PPO1*, a *PPO2* paralogue that is equally enriched in crystal cells, did not recapitulate the phenotype observed using *PPO2* RNAi (Extended Data Fig. 4f–i). Furthermore, the *PPO1*^*Δ*^ or *PPO2*^*Δ*^ null mutants faithfully reproduced the crystal-cell-specific phenotypes observed with *PPO1* or *PPO2* RNAi, respectively (Extended Data Fig. 4g,i).

Collectively, these findings provide strong evidence for an indispensable role of crystal cells in haemocyte translocation. Furthermore, three essential elements within crystal cells—(1) the copper-ion regulators, including Atox1, Ctr1A and MtnA; (2) the expression of CAH2; and (3) the involvement of PPO2—are required for crystal cells to induce haemocyte homing to the haematopoietic pocket.

## O_2_ establishes PPO2 in cellulo crystals

**Fig. 3:**
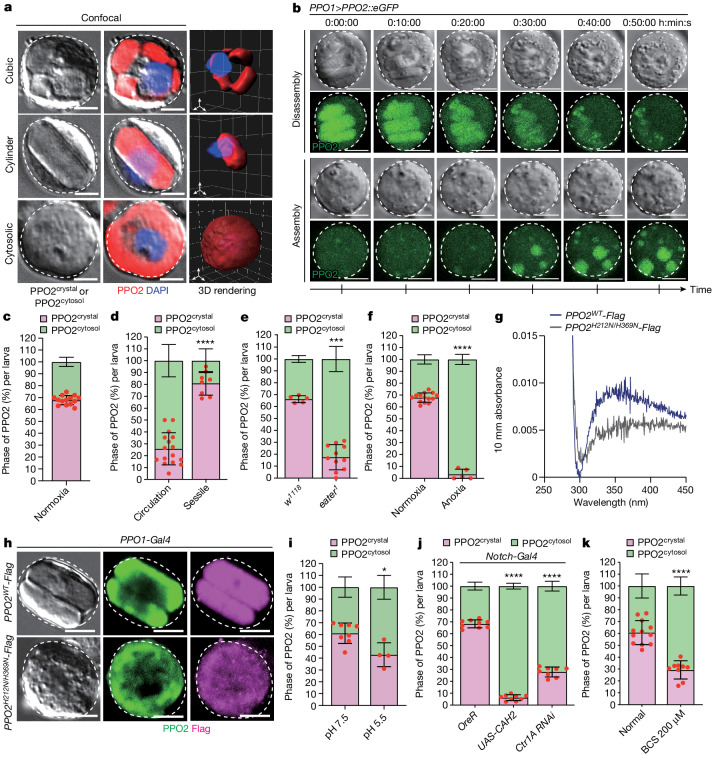
Oxygen, neutral pH and copper are required for the formation of in cellulo PPO2 crystals. **a**,**b**, Structures of in cellulo crystals in crystal cells. **a**, Crystal cells contain crystals that are composed of endogenous PPO2. Crystallized PPO2 was found in cubic (top) or cylindrical shapes (middle), unless localized in the cytosol (bottom). Three-dimensional rendering of confocal images (PPO2, red; DAPI, blue). **b**, Transitions of PPO2 from crystal-to-cytosolic (top) or from cytosolic-to-crystal (bottom) form. Ex vivo live imaging of crystal cells expressing *PPO2-eGFP*. **c**, Quantification of crystalline (magenta) or cytosolic PPO2 (green) in total (sessile and circulation) crystal cells. **d**, Crystal cells in the haematopoietic pocket (sessile) contained a higher ratio of PPO2 crystals compared with those in the haemolymph (circulation). **e**,**f**, Compared with WT *w*^*1118*^, the crystalline PPO2 ratio was reduced in *eater*^*1*^ mutants (**e**) or by culturing larvae under anoxic conditions (0.1% O_2_) for 6 h (**f**). **g**, The absorbance peak indicating copper–oxygen binding at 340 nm was observed with WT PPO2–Flag (blue) but not with PPO2(H212N/H369N)–Flag (grey). **h**, WT PPO2 (PPO2^WT^–Flag) (Flag, magenta) co-localized with endogenous PPO2 (PPO2, green) (top). A PPO2 mutant (PPO2(H212N/H369N)–Flag) (Flag, magenta) was distributed in the cytoplasm and inhibited endogenous PPO2 crystals (PPO2, green). **i**, Crystalline PPO2 from the total crystal cells was reduced by incubating haemocytes in pH 5.5 for 1 h. **j**,**k**, Compared with the controls (left), *CAH2* overexpression (middle), *Ctr1A* RNAi in crystal cells (right) (**j**) or feeding with 200 µM bathocuproine disulfonic acid for 6 h (**k**) reduced the ratio of PPO2 crystals. Scale bars, 5 µm (**a**, **b** and **h**). The single red dots in **c**–**f** and **i**–**k** show the percentage of crystal cells with PPO2^crystal^ per one larva. Statistical analysis was performed using Mann–Whitney *U-*tests (**c**–**f** and **i**–**k**). Data are mean ± s.d. Source Data

**Extended Data Fig. 5:**
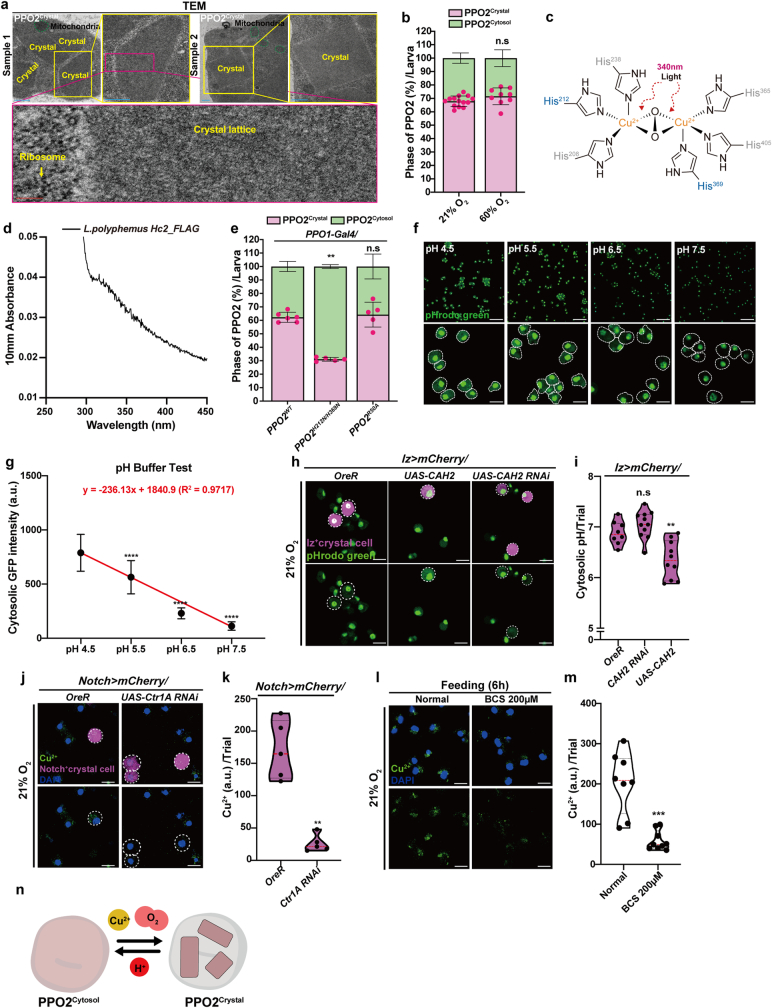
Genetic backgrounds and conditions that manipulate pH or Cu^2+^ levels in hemocytes. (**a**) Ultrastructure of crystal cells observed by transmission electron microscopy (TEM). Sample 1 (left) and sample 2 (right) indicate two independent crystal cells. Yellow insets are magnified on the right. The magenta inset is on the bottom. (**b**) Crystal-to-cytosolic ratio of PPO2 protein in crystal cells at 21% or 60% O_2_. Larvae were bled at 120-h AEL, and entire crystal cells expressing PPO2 were scored. (**c**) Representation of the type III copper centre-containing haemocyanin/tyrosinase domain of PPO2 and the 340-nm absorbance indicating copper–oxygen binding. (**d**) The absorbance peak at 340 nm was detected with *Limulus polyphemus* haemocyanin II (*L.pol* Hc2_FLAG). (**e**) Crystal-to-cytosol ratio of PPO2 protein was reduced by the expression of *PPO2*^*H212N/H369N*^, whereas *PPO2*^*R50A*^, a point mutation outside the copper centre, did not alter the ratio. **f**–**g**. Standard curve analysed with different pH buffers (pH 7.5, pH 6.5, pH 5.5, and pH 4.5) using pHrodo green dye. The lower the pH, the higher the cytosolic green intensity. (**f**) Intensities of pHrodo green dye in hemocytes in different pH conditions (pHrodo green dye, green). (**g**) Quantification graph of cytosolic GFP intensity at different pHs. **h**–**i**. Crystal-cell-specific overexpression of *carbonic anhydrase 2* (*CAH2*) (*lz-Gal4, UAS-mCherry-HA; UAS-CAH2*) lowered pH levels in crystal cells, whereas downregulation of *CAH2* in crystal cells (*lz-Gal4, UAS-mCherry-HA; UAS-CAH2 RNAi*) did not change pH levels at 21% O_2_. (**h**) Intracellular pH levels (pHrodo, green; *lz*^+^ crystal cells, magenta) at 21% O_2_. (**i**) Quantification of intracellular pH concentration in each genetic background. **j**–**k**. Cytosolic Cu^2+^ in the crystal cell was decreased by the crystal-cell-specific knockdown of *Ctr1A*. (**j**) Intracellular Cu^2+^ levels (Cu^2+^ dye, green; *lz*^+^ crystal cells, magenta; DAPI, blue) in wild-type *OreR* controls (left) (*Notch-Gal4 UAS-mCherry*) or *Ctr1A* RNAi (right) (*Notch-Gal4 UAS-mCherry; UAS-Ctr1ARNAi*). (**k**) Quantification of intracellular copper ion levels in controls or *Ctr1A RNAi*. One dot represents one trial. **l**–**m**. Feeding larvae 200 μM bathocuproinedisulfonic acid (BCS) reduced intracellular copper ion concentrations in hemocytes. Larvae were reared in copper chelator-containing food (200 µM BCS) for 6 h from 114-h AEL and dissected at 120-h AEL. (**l**) Intensities of intracellular copper indicator (Cu^2+^, green; DAPI, blue) in the absence (left) or presence (right) of BCS. (**m**) Quantification of intracellular copper ion concentrations in each condition. (**n**) Illustration of the transition from cytosolic-to-crystalline PPO2 associated with intracellular copper, pH, and ambient oxygen. Copper or oxygen promotes the formation of crystallized PPO2. Scale bar: blue, 500 nm; orange, 100 nm; white, 10 µm. n.s (not significant), **P < 0.01, ***P < 0.001, ****P < 0.0001. The single red dots in Extended Data Fig. 5b and e indicate the percentage of crystal cells with PPO2^crystal^ per one larva. Extended Data Fig. 5b, e, g, i, k, and m were analysed using the Mann–Whitney test. Violin plots in **5i**, **5k**, and **5m** denote maximum, 25% (black dotted), median (red dotted), 75% (black dotted), and minimum values, respectively. Error bars in **5b**, **5e**, and **5g** represent standard deviations (SDs). Schematic diagrams in Extended Data Fig. 5n was created with BioRender.com. Source Data

Mature crystal cells contain endogenous crystalline inclusions in their cytoplasm, primarily composed of PPO2 proteins^36,37^. In our study, we confirmed the presence of two distinct groups of mature crystal cells: those with crystals and those without any visible crystals (Fig. 3a). Crystal cells with crystals contained various shapes and sizes of crystalline structures in their cytoplasm, specifically labelled by an antibody targeting endogenous PPO2 (referred to as PPO2^crystal^) (Fig. 3a and Supplementary Videos 1 and 2). Conversely, crystal cells lacking visible crystals showed PPO2 protein localization in the cytoplasm (PPO2^cytosol^) and no evidence of intracellular crystal structures (Fig. 3a and Supplementary Video 3). Transmission electron microscopy (TEM) further visualized uniformly arranged crystal lattice structures within crystal cells, distinguishable from the amorphous, low-density interphase between the crystal and a compact ribosomal array (Extended Data Fig. 5a). Notably, live imaging of crystal cells containing cytosolic PPO2 revealed spontaneous assembly of in cellulo PPO2 crystals within an hour, even without any external stimulation (Fig. 3b and Supplementary Video 4). We also observed the opposite scenario, in which PPO2^crystal^ dissolved into the cytosolic phase during ex vivo culture (Fig. 3b and Supplementary Video 4), suggesting dynamic phase changes of PPO2 within the crystal cell. Through detailed analysis of PPO2 localization in crystal cells under normoxic conditions, we found that 68% of total crystal cells comprised PPO2 as crystals, while the remaining cell population showed PPO2 in the cytoplasm (Fig. 3c). Further examination of PPO2 protein status based on crystal cell localization revealed that sessile crystal cells contained a significantly higher proportion of PPO2^crystal^ (83%), whereas the circulating population displayed only 29% PPO2^crystal^ under normoxia (Fig. 3d). These proportions, compared with those of total crystal cells, with 68% PPO2^crystal^ and 32% PPO2^cytosol^ (Fig. 3c), suggest a biased distribution of crystal cells containing crystallized PPO2 within the haematopoietic pocket.

To investigate the mechanism by which crystal cells generate in cellulo PPO2^crystal^, we used *eater*^*1*^ mutants that eliminate sessile haemocytes, including both plasmatocytes and crystal cells, due to the lack of Nimrod-family scavenger receptors^38^. This genetic background probably interferes with interactions between crystal cells and the trachea as well. Under normoxic conditions, *eater*^*1*^ mutants formed significantly less PPO2^crystal^, with only 16% of crystal cells containing crystallized PPO2 (Fig. 3e). This observation was consistent with the almost complete absence of PPO2 within crystals when larvae were exposed to anoxia (Fig. 3f). By contrast, hyperoxia did not affect the ratio of crystal cells bearing PPO2^crystal^ or PPO2^cytosol^ (Extended Data Fig. 5b). These findings suggest that oxygen availability, probably obtained from the trachea, is critical for the generation of PPO2^crystal^ in crystal cells.

Supporting these findings, the PPO2 protein contains a copper-binding haemocyanin/tyrosinase domain that incorporates two copper ions and oxygen in the type III copper centre alignment^39,40^ (Extended Data Fig. 5c). Oxygenated haemocyanin can be detected by an absorption spectrum peak at around 340 nm, which is characteristic of an oxygen-bound dicopper centre^41^. To validate the oxygen-binding ability of PPO2, we expressed PPO2–Flag in S2R^+^ cells, purified native PPO2 using Flag epitope competition and measured its absorption spectrum. The absorption spectrum of WT PPO2 displayed a peak at 340 nm (Fig. 3g). By contrast, the PPO2(H212N/H369N) mutant protein, containing point mutations in the histidine residues of the dicopper centre, did not exhibit a peak at 340 nm (Fig. 3g). As a positive control, we purified previously characterized oxygen-binding haemocyanin II (*Hc2*; gene ID_106468801) from the Atlantic horseshoe crab *Limulus polyphemus*^42^ and observed an identical peak at 340 nm (Extended Data Fig. 5d). After its expression in crystal cells, PPO2(H212N/H369N) was rarely detected in in cellulo crystals, while simultaneously disrupting the crystallization of endogenous PPO2 (Fig. 3h and Extended Data Fig. 5e). By contrast, the reintroduction of *PPO2*^*R50A*^, bearing a mutation outside the copper-binding centre, did not interfere with PPO2 crystal formation (Extended Data Fig. 5e). These results indicate that O_2_ physically associates with the type III copper centre of PPO2 and is responsible for inducing the formation of PPO2 crystals.

We further investigated the role of intracellular copper and cytosolic pH, regulated by CAH2, in the formation of PPO2^crystal^, considering their significance in haemocyte translocation. To manipulate pH levels in haemocytes, we cultured them ex vivo in solutions of pH 7.5 or 5.5, created by adding valinomycin and nigericin, which facilitate the movement of H^+^ ions across the membrane^43,44^. Compared with at pH 7.5, incubation at pH 5.5 resulted in a reduced proportion of crystal cells containing PPO2^crystal^ at 42% (Fig. 3i and Extended Data Fig. 5f,g). Moreover, when *CAH2* was overexpressed in crystal cells specifically, which leads to acidification of crystal cells (Extended Data Fig. 5h,i), PPO2^crystal^ was dismantled and dispersed throughout the cytoplasm (Fig. 3j). To disrupt homeostatic copper levels in crystal cells, we used *Ctr1A* RNAi and confirmed that crystal-cell-specific *Ctr1A* RNAi reduced copper-ion levels in the crystal cells (Extended Data Fig. 5j,k). In this genetic background, the percentage of crystal cells containing PPO2^crystal^ diminished to 29% (Fig. 3j). Similar results were observed when larvae were cultured with 200 μM bathocuproine disulfonic acid—a copper chelator (Fig. 3k and Extended Data Fig. 5l,m). However, attempts to increase intracellular copper levels were unsuccessful due to immediate copper detoxification by the induction of copper chaperones^45,46^.

Taken together, these findings provide evidence that crystal cells undergo dynamic assembly or disassembly of PPO2 crystals—a process that is modulated by homeostatic copper levels, cytosolic pH controlled by CAH2 and the acquisition of O_2_ through binding to the trachea (Extended Data Fig. 3n).

## Phase transition of PPO2 and oxygenation

**Fig. 4:**
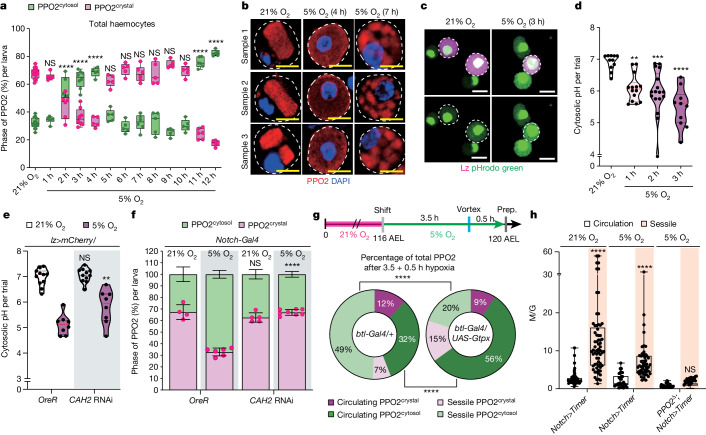
Dynamic phase transitions of PPO2 crystals oxygenate crystal cells. **a**, Quantification of PPO2 protein phase during 12 h hypoxia (PPO2^cytosol^, green; PPO2^crystal^, magenta). **b**, Three representative images of PPO2^+^ crystal cells in normoxia and after 4 h or 7 h hypoxia. **c**, Intracellular pH (pHrodo, green; lz^+^ crystal cells, magenta) in normoxia (left) or after 3 h hypoxia (right). **d**, Quantification of the intracellular pH of crystal cells shown in **c**. **e**, Quantification of the intracellular pH in *OreR* controls or *CAH2* RNAi flies. **f**, Crystal-cell-specific *CAH2* RNAi rescued the low pH levels seen at 3 h hypoxia. Quantification of the PPO2^crystal^ and PPO2^cytosol^ ratio in control or *CAH2* RNAi flies under normoxia or hypoxia (PPO2^cytosol^, green; PPO2^crystal^, magenta). **g**, A higher proportion of PPO2^cytosol+^ crystal cells adhered to the haematopoietic pocket after a 30 min reattachment assay (left). PPO2^cytosol^-mediated adherence was inhibited by scavenging H_2_O_2_ (right). **h**, Quantification of the M/G *nlsTimer* ratio in controls or in *PPO2*^*Δ*^ mutants corresponding to Extended Data Fig. 6n. Red fluorescence of *nlsTimer* was converted into magenta to avoid the red/green colour scheme. Scale bars, 5 µm (**b**) and 10 µm (**c**). The magenta dots in **a** and **f** show the percentage of crystal cells with PPO2^crystal^ per individual larva. Statistical analysis was performed using one-way ANOVA followed by Tukey’s post hoc analysis (**a** and **d**), two-way ANOVA followed by Bonferroni’s post hoc analysis (**e**–**g**) and Mann–Whitney *U*-tests (**h**). For **a** and **h**, the box and whisker plots show the median (centre line), 25% and 75% (box limits), and the maximum and minimum values (whiskers). For **d** and **e**, the violin plots show the median (red dotted line), 25% (black dotted line), 75% (black dotted line), and the maximum and minimum values. For **f**, data are mean ± s.d. Detailed genotypes, sample sizes, statistics and *Gal4* drivers are provided in Supplementary Table 5. Source Data

**Extended Data Fig. 6:**
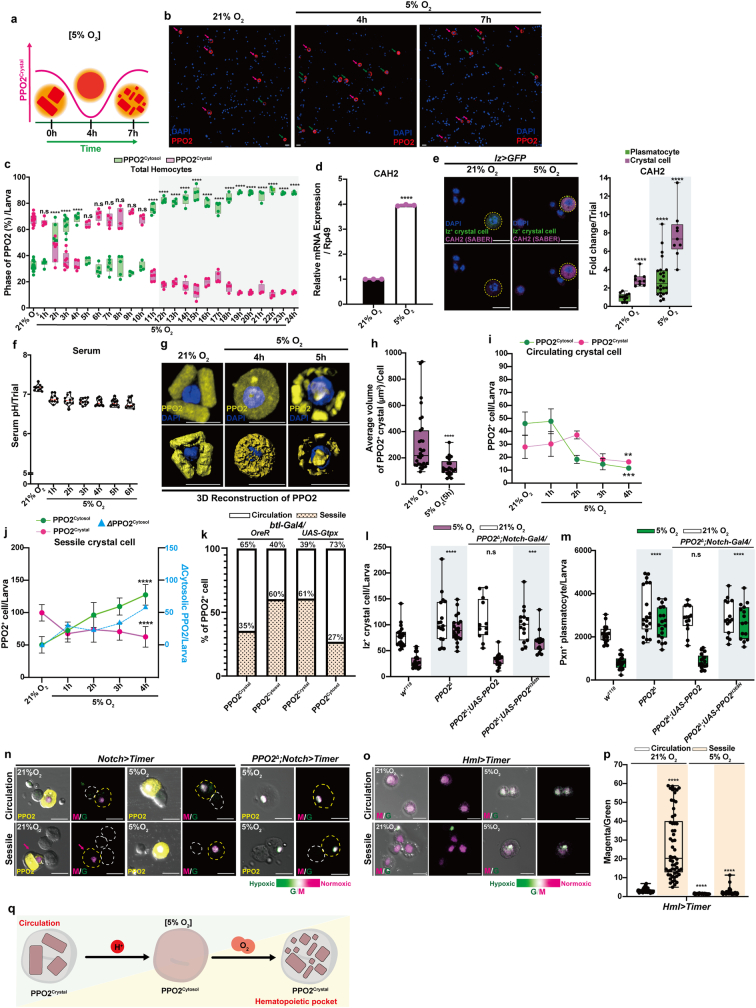
Changes in crystal-to-cytosolic phase transitions of PPO2 during 24-h hypoxia. (**a**) Illustration of the phase transition of PPO2 under hypoxia. (**b**) Representative images of circulating hemocytes with PPO2^+^ crystal cells at each time point. Red arrows indicate crystalline PPO2, and green arrows indicate the cytosolic form of PPO2 (PPO2^+^ crystal cells, red; DAPI, blue). In 21% O_2_, most PPO2^+^ cells contained PPO2 in its crystalline state (left). During 4 h in hypoxia, the majority of PPO2^+^ cells contained PPO2 in its cytosolic form (middle). During 7 h in hypoxia, crystal cells contained smaller crystals of PPO2 (right). (**c**) Crystal-to-cytosolic ratio of PPO2 protein in crystal cells changed during hypoxia up to 24 h. Quantification of PPO2 protein distribution patterns (cytosolic PPO2, green; crystalline PPO2, magenta) at each time point. **d**–**e**. The level of *CAH2* mRNA was induced by 3-h hypoxia. (**d**) Relative mRNA expression levels of *CAH2* in normoxia and 3-h hypoxia. (**e**) Visualization of *CAH2* mRNA by SABER-FISH. lz^+^ crystal cells (lz, green) already exhibited higher *CAH2* levels (CAH2, magenta; dotted yellow circle) than plasmatocytes (DAPI^+^) in normoxia, which was further enhanced by hypoxia. Quantification of *CAH2* levels is shown on the right. (**f**) Haemolymph pH levels remained relatively constant in hypoxia. Synchronized larvae (*w*^*1118*^) were reared in 21% O_2_ and transferred to hypoxia for the indicated amounts of time, and haemolymph was extracted at 120-h AEL. (**g**) Three-dimensional reconstruction of PPO2 protein phases (PPO2^+^ crystal cells, yellow; DAPI, blue) at 21% O_2_ (crystalline PPO2, left), in 4 h of culture in hypoxia (cytosolic PPO2, middle), or 5 h of culture in hypoxia (fractionated crystalline PPO2, right). (**h**) Recrystallization reduced the average volume of PPO2^+^ crystals per one crystal cell following 5-h hypoxia compared with intact PPO2 crystals generated in controls. (**i**) Quantification of crystalline or cytosolic PPO2 in the circulation. The numbers of cytosolic PPO2 (green) and crystalline PPO2 (magenta) gradually decreased until 4-h hypoxia. (**j**) Quantification of crystal cells containing crystalline or cytosolic PPO2 in the hematopoietic pocket (sessile) during 4 h of hypoxia. ΔCytosolic PPO2 indicates changes in the number of PPO2^cytosol^ per one larva (light blue, Y axis on the right). (**k**) The ratios of crystal cell sessility classified based on the PPO2 protein status shown in Fig. 4g. **l**–**m**. Oxygen binding capacity of PPO2 is critical for the hemocyte movement during hypoxia. Reintroduction of *PPO2*^*WT*^ into the *PPO2*^*Δ*^ mutant rescued hemocyte movement during 4-h hypoxia, while *PPO2*^*H369N*^ did not (*PPO2*^*Δ*^*; Notch-Gal4 UAS-PPO2*^*WT*^ or *UAS-PPO2*^*H369N*^). Quantification of lz^+^ crystal cells (**l**) or Pxn^+^ plasmatocytes (**m**). (**n**) Expression of crystal-cell-specific *nlsTimer* in controls (*Notch-Gal4 UAS-nlsTimer*) or in *PPO2*^*Δ*^ mutants (*PPO2*^*Δ*^*; Notch-Gal4 UAS-nlsTimer*) (PPO2^+^ crystal cells, magenta; nlsTimer, red/green merged) in the circulation (top) or in the hematopoietic pocket (bottom) in normoxic or hypoxic conditions, corresponding to Fig. 4h. **o**–**p**. Plasmatocytes exhibited higher magenta-to-green nlsTimer ratios in the hematopoietic pocket than in the circulation (*Hml*^*Δ*^*-Gal4 UAS-nlsTimer*). (**o**) Maturation of plasmatocyte-specific nlsTimer (magenta-to-green merged) in circulating cells (top) or sessile cells (bottom) at 21% O_2_ (left) or 5% O_2_ (right). (**p**) Quantification of the magenta/green ratio for each condition corresponding to panel (**o**). Red fluorescence of nlsTimer was converted into magenta to avoid the red/green colour scheme. (**q**) Illustration of the PPO2 crystal-to-cytosol transition upon hypoxia. Scale bar, 10 µm. n.s (not significant), **P < 0.01, ***P < 0.001, ****P < 0.0001. The single red dots in Extended Data Fig. 6c indicate the percentage of crystal cells with PPO2^crystal^ per one larva. Extended Data Fig. 6c was analysed by one-way ANOVA followed by Tukey’s post hoc test. Extended Data Fig. 6d, e, h, i, and p were analysed using the Mann–Whitney test. Extended Data Fig. 6l and m was analysed by two-way ANOVA followed by Dunnett’s multiple comparison test. Box and whiskers plots in **6c**, **6e**, **6h**, **6l**, **6m**, and **6p** denote maximum, 25%, median, 75%, and minimum values, respectively. Error bars in **6d**, **6i**, and **6j** represent standard deviations (SDs). Schematic diagrams in Extended Data Fig. 6q was created with BioRender.com. Source Data

During the assessment of the PPO2^crystal^ phase over a 24 h period of hypoxic stress (Methods), we observed a gradual decrease in the proportion of PPO2^crystal^ starting at 2 h of exposure, ultimately reaching a low of 31% after 4 h of hypoxia (Fig. 4a,b and Extended Data Fig. 6a–c). Within 3 h of hypoxia, crystal cells showed a decrease in cytosolic pH as well as a significant increase in *CAH2* mRNA levels (Fig. 4c,d and Extended Data Fig. 6d,e), which could be neutralized by *CAH2* RNAi in the crystal cells (Fig. 4e). Furthermore, crystal-cell-specific knockdown of *CAH2* prevented the dissolution of PPO2^crystal^ (Fig. 4f), which correlated with the absence of haemocyte translocation (Fig. 2e). While the intracellular pH of crystal cells changed during hypoxia, the pH of the haemolymph remained relatively constant (Extended Data Fig. 6f). After the initial breakdown, the fraction of PPO2^crystal^ began to increase again, reaching 61% after 5 h culture in hypoxia (Fig. 4a,b and Extended Data Fig. 6a–c). The proportion of PPO2^crystal^ returned to WT levels after 5 to 10 h of hypoxia, exhibiting a damped oscillatory-like pattern similar to the pattern of the numbers of circulating crystal cells (Figs. 1g and 4a and Extended Data Fig. 6a–c). The recycled PPO2^crystal^ observed at later timepoints appeared smaller in size compared with those of the controls (Fig. 4b and Extended Data Fig. 6g,h) and eventually dissolved back into the cytosolic phase after 11 h, resulting in a stationary phase (Extended Data Fig. 6c).

Based on the observed dynamics of PPO2^crystal^ and PPO2^cytosol^ in crystal cells during hypoxia, we hypothesized that the protein phase transition of PPO2 has a crucial role in the crystal-cell-mediated homing of plasmatocytes and crystal cells to the haematopoietic pocket. During 4 h under hypoxia, a notable change occurred in the distribution of PPO2^cytosol^ and PPO2^crystal^ in crystal cells. PPO2^cytosol^ disappeared from circulation and accumulated in the haematopoietic pocket, while PPO2^crystal^ decreased, both in circulation and the haematopoietic pocket (Extended Data Fig. 6i,j). The change in the number of crystal cells containing PPO2^cytosol^ (ΔPPO2^cytosol^) in the haematopoietic pocket was more pronounced compared with ΔPPO2^cytosol^ in circulation, suggesting that both PPO2^cytosol^ in circulation and dissolved PPO2^crystal^ probably contribute to ΔPPO2^cytosol^ in the haematopoietic pocket (Extended Data Fig. 6j). To further investigate this phenomenon, we examined whether crystal cells containing PPO2^cytosol^ preferentially relocate to the haematopoietic pocket. After 3.5 h of exposure to hypoxia, haemocytes were physically deattached and allowed to reattach to the haematopoietic pocket for 0.5 h (Methods). Of the total crystal cells, 56% of PPO2^+^ crystal cells reattached to the haematopoietic pocket within 30 min. Among these, 49% contained PPO2^cytosol^, while only 7% contained PPO2^crystal^ (Fig. 4g). When considering PPO2^crystal^ and PPO2^cytosol^ independently, 60% PPO2^cytosol^ and 35% PPO2^crystal^ returned to the haematopoietic pocket, while 40% PPO2^cytosol^ and 65% of PPO2^crystal^ remained in circulation (Fig. 4g and Extended Data Fig. 6k). Conversely, scavenging H_2_O_2_ in the trachea by overexpression of *Gtpx* significantly reduced the reattachment rate of PPO2^+^ crystal cells to 35%, of which only 20% was PPO2^cytosol^ (Fig. 4g and Extended Data Fig. 6k). The reintroduction of *PPO2*^*H369N*^, containing a mutation in the dicopper centre, into *PPO2*^*Δ*^-mutant crystal cells did not restore the localization of crystal cells and plasmatocytes after 4 h hypoxia (Extended Data Fig. 6l,m). These findings suggest that crystal cells containing PPO2^cytosol^ with oxygen-binding ability preferentially translocate to the haematopoietic pocket through H_2_O_2_ derived from the trachea.

To investigate the role of PPO2 in crystal cell oxygenation associated with adherence to the trachea, we used *nlsTimer*, a fluorescent protein variant that measures oxygen availability. Mature *nlsTimer* competitively emits green or red fluorescence, but red fluorescence maturation (red is indicated in magenta in this study) is highly dependent on oxygen concentration^47^. Under normoxic conditions at 21% O_2_, plasmatocytes and crystal cells in the haematopoietic pocket displayed higher oxygen levels compared with those in circulation, as indicated by a higher magenta-to-green (M/G) ratio (Fig. 4h and Extended Data Fig. 6n). Under hypoxic conditions, both sessile and circulating plasmatocytes showed significantly reduced M/G ratios, indicating lower oxygen availability regardless of their location (Extended Data Fig. 6o,p). However, mature crystal cells expressing PPO2 exhibited higher magenta fluorescence levels in the haematopoietic pocket compared with those in circulation, even under hypoxic conditions (Fig. 4h and Extended Data Fig. 6n). By contrast, *PPO2*^*Δ*^ mutant crystal cells did not show higher magenta fluorescence levels in the haematopoietic pocket and displayed a similar M/G ratio to circulating crystal cells under hypoxic conditions (Fig. 4h and Extended Data Fig. 6n), suggesting that PPO2 promotes crystal cell oxygenation.

In summary, these results suggest that, under hypoxic conditions, the combination of low ambient O_2_ and CAH2-mediated reduction in cytosolic pH in crystal cells leads to the dissolution of the crystalline phase of PPO2 protein into its cytosolic form. This process facilitates the translocation of crystal cells to the haematopoietic pocket and supports oxygenation and recrystallization of PPO2 (Extended Data Fig. 6q). The phase and location transition of PPO2 probably serve as an oxygen reservoir, contributing to animal respiration and the maintenance of oxygen homeostasis.

## PPO2 supports internal O_2_ homeostasis

**Extended Data Fig. 7:**
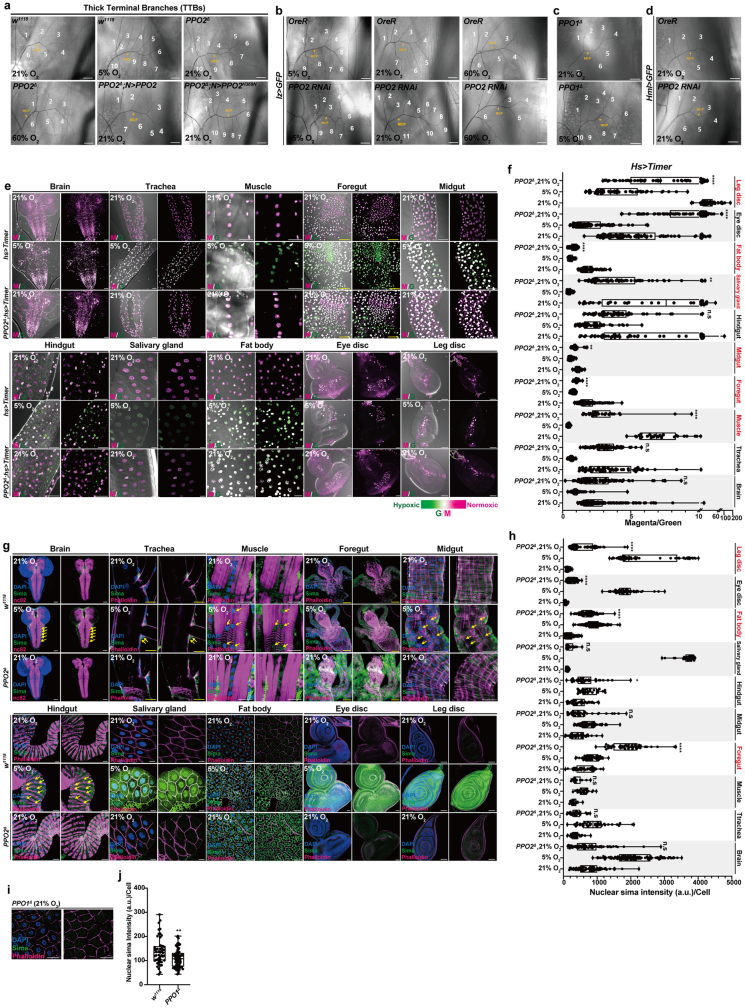
A lack of crystal cells or *PPO2* leads to internal hypoxia. (**a**) Tracheal thick terminal branches (TTBs) were increased in wild type (*w*^*1118*^) under hypoxia or in *PPO2*^*Δ*^ mutants under normoxia. Culturing *PPO2*^*Δ*^ mutant larvae in hyperoxia or reintroducing *PPO2* in *PPO2*^*Δ*^ mutant crystal cells (*PPO2*^*Δ*^*; Notch-Gal4 UAS-PPO2*) rescued TTB numbers (TTB numbers, white; main cellular process (MCP), yellow). Reintroducing the mutant form of PPO2 in *PPO2*^*Δ*^ mutant crystal cells (*PPO2*^*Δ*^*; Notch-Gal4 UAS-PPO2*^*H369N*^) did not rescue TTB numbers. Quantification of TTB numbers in these genetic backgrounds or conditions is presented in Supplementary Tables 1 and 2. (**b**) Crystal-cell-specific downregulation of *PPO2* (*lz-Gal4 UAS-EGFP UAS-PPO2 RNAi*) exhibited increased tracheal thick terminal branching (TTB) compared with wild-type (*lz-Gal4 UAS-EGFP*), which was recovered by culturing larvae in hyperoxia. (**c**) *PPO1*^*Δ*^ mutants or (**d**) plasmatocyte-specific downregulation of *PPO2* (*Hml*^*Δ*^*-Gal4 UAS-EGFP UAS-PPO2 RNAi*) did not recapitulate the increased TTB development observed in *PPO2*^*Δ*^ or *PPO2* RNAi crystal cells (*lz-Gal4 UAS-EGFP UAS-PPO2 RNAi*). Quantification of TTB numbers in these genetic backgrounds or conditions are shown in Supplementary Tables 1 and 2. **e**–**i**. Oxygenation levels of the brain, trachea, muscle, foregut, midgut, and hindgut of the intestine, salivary gland, fat body, eye disc, and leg disc measured by *nlsTimer* or nuclear sima. (**e**) Wild-type larvae (*hs-gal4 UAS-nlsTimer*) were synchronized and transferred to 5% O_2_ at 66-h AEL for 54 h after heat shock. Expression of *nlsTimer* (magenta and green merged) control tissues (*hs-gal4 UAS-nlsTimer*) at normoxia (top), hypoxia (middle), or *PPO2*^*Δ*^ mutant tissues (*PPO2*^*Δ*^*; hs-gal4 UAS-nlsTimer*) at normoxia (bottom). (**f**) Quantification of the magenta-to-green ratio in each condition and genetic background shown in (**e**). Red fluorescence of nlsTimer was converted into magenta to avoid the red/green colour scheme. (**g**) Expression and localization of nuclear sima in wild-type or *PPO2*^Δ^ mutant tissues, corresponding to (**e**). Wild-type *w*^*1118*^ tissues at normoxia (top), hypoxia (middle), or *PPO2*^*Δ*^ mutant tissues at normoxia (bottom). (**h**) Quantification of nuclear sima in each condition and genetic background in (**g**). **i**–**j**. Fat body nuclear sima levels in *PPO1*^*Δ*^ mutants remained unchanged compared with wild-type *w*^*1118*^. (**i**) Expression and localization of sima (sima, green; phalloidin, magenta; DAPI, blue) in the fat body. (**j**) Quantification of nuclear sima levels in the fat body in each genetic background. Scale bars: yellow, 10 µm; white, 50 µm. n.s (not significant), **P < 0.01, ***P < 0.001, ****P < 0.0001. Extended Data Fig. 7f, i, and k were analysed using the Mann–Whitney test. Box and whiskers plots in **7f**, **7h**, and **7j** denote maximum, 25%, median, 75%, and minimum values, respectively. Tissues marked in red (Y axis on the right) indicate relatively low M/G (**f**) or high nuclear sima levels (**h**). Source Data

Similar to *lz*^*r15*^ mutants, *PPO2*^*Δ*^ mutants and larvae carrying *PPO2* RNAi in crystal cells exhibited a higher number of TTBs under normoxic conditions (Extended Data Fig. 7a,b and Supplementary Tables 1 and 2). This phenotype was not exacerbated by hypoxia (Extended Data Fig. 7a,b and Supplementary Tables 1 and 2). Moreover, hyperoxic conditions again rescued the increased TTBs phenotype in *PPO2*^*Δ*^ mutant larvae, restoring TTB numbers to WT levels (Extended Data Fig. 7a and Supplementary Tables 1 and 2). Reintroduction of WT PPO2 into *PPO2*^*Δ*^ mutants rescued the TTBs phenotype, whereas *PPO2*^*H369N*^, which lacks the ability to bind to oxygen, did not reduce the number of TTBs (Extended Data Fig. 7a and Supplementary Tables 1 and 2). In contrast to *PPO2*^*Δ*^, *PPO1*^*Δ*^ mutants displayed only a marginal increase in TTBs under normoxia, which was further increased by hypoxia (Extended Data Fig. 7c and Supplementary Tables 1 and 2). *PPO2* RNAi in plasmatocytes did not alter TTB numbers (Extended Data Fig. 7d and Supplementary Tables 1 and 2).

**Fig. 5:**
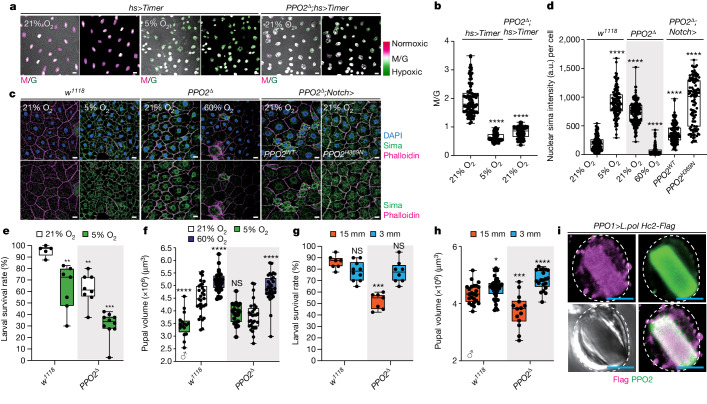
PPO2 in crystal cells maintains internal oxygen homeostasis. **a**,**b**, Oxygenation levels of the fat body measured by *nlsTimer*. The fat body was hypoxic when larvae were raised under hypoxia or if they carried the *PPO2* mutant. **a**, Expression of *nlsTimer* (M/G merged) in the fat body. **b**, Quantification of the M/G ratios for each condition and genetic background. Red fluorescence of *nlsTimer* was converted into magenta to avoid the red/green colour. **c**, WT larvae (*w*^*1118*^) cultured in hypoxia or *PPO2*^*Δ*^ mutants under normoxia accumulated nuclear sima in the fat body, which was rescued by the reintroduction of *PPO2*^*WT*^, but not by *PPO2*^*H369N*^ (sima, green; phalloidin, magenta; DAPI, blue). **d**, Quantification of nuclear sima levels in WT or *PPO2*^*Δ*^ flies, or *PPO2*^*Δ*^ flies with reintroduction of *PPO2*^*WT*^ or *PPO2*^*H369N*^. **e**, PPO2 is required for larval survival. Larval survival rates of WT or *PPO2*^*Δ*^ mutants under normoxic or hypoxic conditions. **f**, *PPO2*^*Δ*^ mutants had reduced pupal sizes, which were recovered by hyperoxia. Quantification of the male pupal volume in WT or *PPO2*^*Δ*^ mutant animals in normoxic, hypoxic or hyperoxic conditions. **g**, The reduced larval survival rate of the *PPO2*^*Δ*^ mutant was rescued by 3-mm-depth food culture. **h**, *PPO2*^*Δ*^ mutants caused reduced animal growth, which was recovered in 3-mm-depth food. Quantification of the male pupal volume in WT or *PPO2*^*Δ*^ mutant animals cultured in 15 mm or 3 mm food depths. **i**, *L. polyphemus* haemocyanin II formed crystalline structures that co-localized with endogenous PPO2 crystals (Hc2–Flag, magenta; PPO2, green). Scale bars, 10 µm (**a** and **c**) and 5 µm (**i**). Statistical analysis was performed using Mann–Whitney *U*-tests (**b** and **d**–**h**). For **b** and **d**–**h**, the box and whisker plots show the median (centre line), 25% and 75% (box limits), and the maximum and minimum values (whiskers). White bars, normoxia; green bars, hypoxia; purple bars, hyperoxia; orange bars, 15-mm-depth food; blue bars, 3-mm-depth food. Source Data

Under normoxic conditions, various WT tissues expressing *nlsTimer*, including the fat body, brain, trachea, muscle, salivary gland, imaginal discs and intestines, uniformly displayed high M/G ratios, indicative of high oxygenation levels (Fig. 5a,b and Extended Data Fig. 7e,f). However, hypoxia significantly reduced the overall M/G ratios in these tissues compared with normoxia^47^ (Fig. 5a,b and Extended Data Fig. 7e,f). In *PPO2*^*Δ*^ mutants, the relative M/G ratios of most organs were comparable to those in WT animals under normoxic conditions (21% O_2_) (Extended Data Fig. 7e,f). Notably, the fat body of *PPO2*^*Δ*^ mutants exhibited significantly lower M/G ratios, similar to those in hypoxic WT tissues (Fig. 5a,b). To further confirm the oxygenation levels of the fat body, we examined the protein expression and localization of hypoxia-inducible factor 1α (HIF-1α, sima in *Drosophila*), a representative hypoxia-inducible transcription factor in animals^48^. Similar to the reduced M/G ratio observed in *nlsTimer*, hypoxia stabilizes and induces the nuclear translocation of sima in *Drosophila*. Indeed, hypoxia triggered accumulation of nuclear sima in the fat body, whereas sima was very low in the WT tissues, including the fat body under normoxic conditions (Fig. 5c,d and Extended Data Fig. 7g,h). Consistent with the *nlsTimer* results, *PPO2*^*Δ*^ mutants exhibited higher nuclear sima levels in the fat body under normoxia that returned to WT levels under hyperoxia (Fig. 5c,d). This phenotype was not observed in *PPO1*^*Δ*^ mutants (Extended Data Fig. 7i,j). Moreover, reintroduction of WT *PPO2* into the crystal cells of *PPO2*^*Δ*^ mutants restored low sima protein expression in the fat body, whereas *PPO2*^*H369N*^ did not reduce the elevated nuclear sima levels (Fig. 5c,d). In addition to the fat body, imaginal discs of appendages and the foregut in *PPO2*^*Δ*^ mutants exhibited relatively lower *nlsTimer* M/G ratios and higher nuclear sima intensities under normoxia compared with their WT counterparts (Extended Data Fig. 7e–h).

In conclusion, we propose that PPO2 in crystal cells has a crucial role in promoting tissue oxygenation and maintaining internal oxygen homeostasis. This is supported by its effects on tracheal branching, the *nlsTimer* hypoxia sensor and nuclear sima localization. Specifically, PPO2 in crystal cells wields the most influence over fat body oxygenation during larval development.

## Crystal-cell-mediated larval respiration

**Extended Data Fig. 8:**
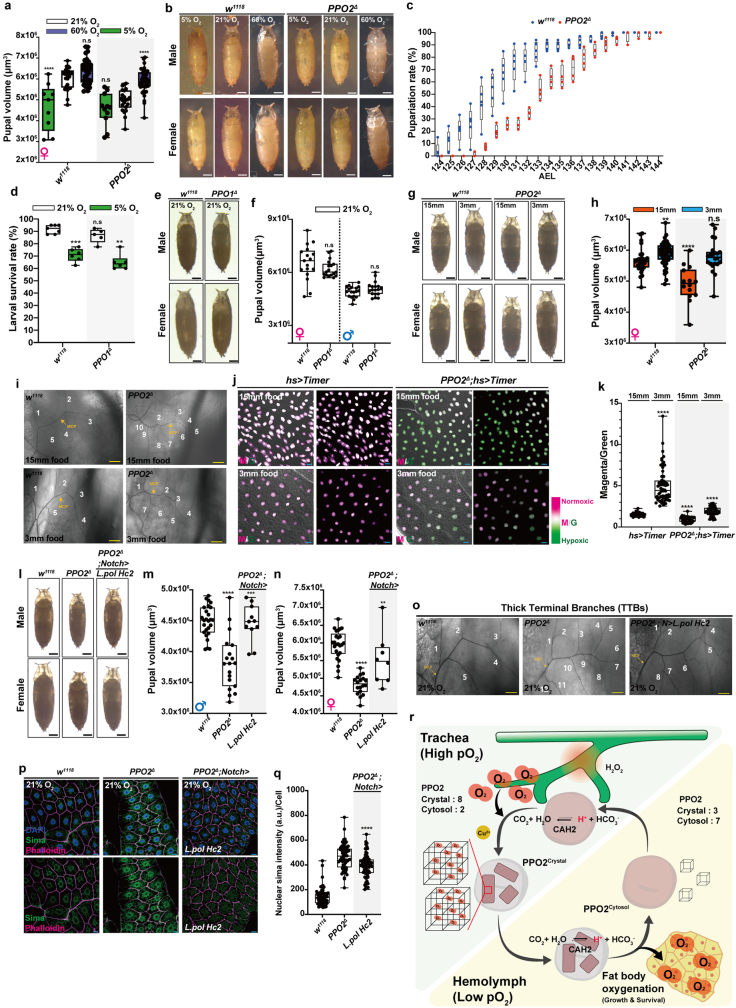
PPO2 in the crystal cell controls animal growth, and shallow food or *Limulus polyphemus haemocyanin II* rescues internal hypoxia in PPO2^*Δ*^ mutants. **a**–**b**. *PPO2*^*Δ*^ mutants had reduced pupal sizes, which were recovered by growing them in hyperoxia. (**a**) Quantitation of female pupal volume in wild-type (*w*^*1118*^) or *PPO2*^*Δ*^ mutant animals in normoxic, hypoxic, or hyperoxic (purple) conditions. (**b**) PPO2^*Δ*^ mutants in normoxia exhibited smaller pupal sizes than wild-type controls (*w*^*1118*^), which was comparable to wild-type controls (*w*^*1118*^) cultured in hypoxia. The smaller pupal sizes of *PPO2*^*Δ*^ mutants were rescued by culturing larvae in hyperoxia. Representative pupal images corresponding to Fig. 5f and Extended Data Fig. 8a. (**c**) Compared with wild-type controls (*w*^*1118*^), the pupariation rate was delayed in the *PPO2*^*Δ*^ mutant background (*w*^*1118*^, blue dot; *PPO2*^*Δ*^, red dot) (**d**) *PPO1*^*Δ*^ mutants did not exhibit altered larval survival. Larval survival rates of wild-type control (*w*^*1118*^) or *PPO1*^*Δ*^ mutant larvae in normoxic (white) or hypoxic (green) conditions. **e**–**f**. Pupal sizes of *PPO1*^*Δ*^ mutants remained unchanged compared with wild-type controls (*w*^*1118*^). (**e**) Representative pupal images corresponding to panel (**f**). (**f**) Quantification of pupal volume in each genetic background. **g**–**h**. The smaller pupal sizes in *PPO2*^*Δ*^ were rescued by providing shallow food at a depth of 3 mm. (**g**) Representative pupal images corresponding to Fig. 5h and Extended Data Fig. 8h. (**h**) Quantification of female pupal volume in wild-type (*w*^*1118*^) or *PPO2*^*Δ*^ mutant animals cultured in 15-mm or 3-mm food depths. (**i**) Increased TTB numbers of *PPO2*^*Δ*^ mutant larvae were rescued by shallow food at a 3-mm depth. Representative TTB images, corresponding to Supplementary Tables 1 and 2. **j**–**k**. Shallow food at a 3-mm depth rescued hypoxic *nlsTimer* expression in *PPO2*^*Δ*^ mutant larvae. (**j**) Maturation of *nlsTimer* (magenta/green merged) in wild-type (*hs-gal4 UAS-nlsTimer*) or *PPO2*^*Δ*^ mutant (*PPO2*^*Δ*^*; hs-gal4 UAS-nlsTimer*) fat bodies. (**k**) Quantification of the magenta/green ratio in each condition and genetic background. Red fluorescence of nlsTimer was converted into magenta to avoid the red/green colour scheme. **l**–**n**. The smaller pupal sizes observed in *PPO2*^*Δ*^ mutants were rescued by reintroduction of *Limulus polyphemus haemocyanin II* (*L. pol Hc2*) in the crystal cell. (**l**) Representative pupal images corresponding to Extended Data Fig. 8m, n. (**m**) Quantification of male pupal volume in controls (*w*^*1118*^), *PPO2*^*Δ*^ mutants, or *PPO2*^*Δ*^*; Notch-Gal4 UAS-L.pol Hc2-FLAG* in normoxia. (**n**) Quantification of female pupal volume in controls (*w*^*1118*^), *PPO2*^*Δ*^ mutants, or *PPO2*^*Δ*^*; Notch-Gal4 UAS-L.pol Hc2-FLAG* mutants in normoxia. (**o**) Reintroducing *L. polyphemus haemocyanin II* in the crystal cell (*PPO2*^*Δ*^*; Notch-Gal4 UAS-L.pol Hc2-FLAG*) rescued TTB numbers. Quantification of TTBs in these genetic backgrounds is shown in Supplementary Tables 1 and 2. **p**–**q**. Reintroducing *L. polyphemus haemocyanin 2* in the crystal cell (*PPO2*^*Δ*^*; Notch-Gal4 UAS-L.pol Hc2-FLAG*) rescued increased sima expression in the *PPO2*^*Δ*^ mutant fat body. (**p**) Expression and localization of nuclear sima (sima, green; phalloidin, magenta; DAPI, blue) in the fat body. (**q**) Quantification of nuclear sima expression in the fat body in each genetic background. (**r**) PPO2 in the crystal cell controls internal oxygen homeostasis and undergoes a phase transition between a crystalline form that acquires oxygen from the trachea and a cytosolic form that releases oxygen to oxygenate the fat body. Scale bars: yellow, 50 µm; white and black, 500 µm; blue, 10 µm. n.s (not significant), **P < 0.01, ****P < 0.0001. Extended Data Fig. 8a, d, f, h, k, m, n, and q were analysed using the Mann–Whitney test. Highest and lowest bars denote maximum and minimum values, respectively. White bars indicate 21% O_2_, and green bars indicate 5% O_2_. Box and whiskers plots in Extended Data Fig. 8 denote maximum, 25%, median, 75%, and minimum values, respectively. Schematic diagrams in Extended Data Fig. 8p was created with BioRenders.com. Source Data

We observed that only 61% of *PPO2*^*Δ*^ mutants survived to the third instar, which is equivalent to the survival rate of *lz*^*r15*^ mutant larvae cultured in normoxia (Fig. 5e). When cultured in hypoxia, the survival rate of *PPO2*^*Δ*^ mutant larvae further decreased to 33% (Fig. 5e), indicating a critical role for PPO2 in crystal cells for animal survival under all conditions. In addition to the survival rate, *PPO2*^*Δ*^ mutants exhibited smaller pupal sizes compared with WT animals under normoxia, comparable to the pupal sizes of WT animals cultured in hypoxia (Fig. 5f and Extended Data Fig. 8a,b). Hyperoxia restored the pupal sizes of *PPO2*^*Δ*^ mutants to WT levels in both males and females (Fig. 5f and Extended Data Fig. 8a,b). Moreover, *PPO2*^*Δ*^ mutants showed delayed pupariation (Extended Data Fig. 8c). *PPO1*^*Δ*^ mutants did not exhibit reduced larval survival or pupal sizes (Extended Data Fig. 8d–f), consistent with our observations on the effects of *PPO1* loss (Extended Data Fig. 7c,i,j and Supplementary Tables 1 and 2). Furthermore, recovery experiments using food at a depth of 3 mm rescued the *PPO2*^*Δ*^ mutant phenotypes, including the decreased survival rates, small pupal sizes (Fig. 5g,h and Extended Data Fig. 8g–h), decreased TTBs (Extended Data Fig. 8i and Supplementary Tables 1 and 2) and the lower M/G ratios of *nlsTimer* (Extended Data Fig. 8j,k).

Finally, prompted by the detection of oxyhaemocyanin by the absorption spectrum at 340 nm (Extended Data Fig. 5d), we investigated whether the haemocyanin protein from the Atlantic horseshoe crab *L. polyphemus* could replace PPO2 in *Drosophila*. Notably, overexpression of *Hc2* from *L. polyphemus* in crystal cells was sufficient to generate in cellulo crystals that colocalized with PPO2 (Fig. 5i). Furthermore, the expression of *L. polyphemus Hc2* in *PPO2*^*Δ*^*-*mutant crystal cells rescued the hypoxic phenotypes observed in *PPO2*^*Δ*^ mutants, including the accumulation of nuclear sima in the fat body, increased TTB numbers and smaller pupal sizes (Extended Data Fig. 8l–q). These findings suggest that haemocyanin protein from other arthropods can substitute for the respiratory function of *Drosophila* PPO2.

Taken together, our observations demonstrate that the respiratory function of PPO2 in crystal cells is essential for larval growth and survival (Extended Data Fig. 8r).

## Discussion

Here we present evidence for the critical role of *Drosophila* haemocytes, specifically crystal cells, in insect respiration through the phase transition of PPO2. Crystal cells orchestrate the dynamic shuttling of haemocytes between the haematopoietic pocket and the circulation. This movement is essential for generating in cellulo crystals of PPO2, which is primarily contained within crystal cells and undergoes phase transitions according to oxygen levels, intracellular pH and copper concentrations. As a result, the ambient oxygen level is a key determinant of the status of PPO2 crystals and substantially influences the duration of crystal cell adherence to the haematopoietic pocket. The phase transition of PPO2 enables it to either be oxygenated and crystallize, serving as a potential oxygen reservoir, or deoxygenate and dissolve to support the respiratory process. Notably, this process mainly targets the fat body—a critical regulator of animal growth and survival that is highly dependent on oxygen availability with insufficient tracheal innervation^28,49^. Consequently, larvae lacking crystal cells or PPO2 or expressing a mutant form of PPO2 incapable of binding to copper ions display hypoxic responses under normoxic conditions and become intolerant to hypoxia. Notably, our results demonstrate that the expression of Hc2 from *L. polyphemus* in crystal cells effectively restores the respiratory function of PPO2, underscoring the evolutionary conservation and significance of this mechanism in respiration.

The presence of membraneless organelles, mainly characterized by liquid–liquid phase separation, has become increasingly recognized in various cellular processes^50–52^. Building on these discoveries, our study reveals the reversible phase transition of PPO2 within crystal cells as a critical regulator of animal respiration. Under normoxic conditions, crystal cell relocation and PPO2 phase transition probably occur while maintaining dynamic equilibrium. This process facilitates the respiration of tissues that lack tracheal innervation or exhibit high oxygen demand, including but not limited to the fat body. While cytosolic PPO1 is known for its role in the main phenoloxidase activities, and crystallized PPO2 for its role in the sequestration of enzymatic phenoloxidase functions^36^, our study indicates an additional function of PPO2 protein in reversible oxygen collection. Notably, our findings demonstrate that haemocyanin from the horseshoe crab co-constitutes crystals with PPO2 (Fig. 5i). Moreover, haemoglobin solutions have been observed to undergo phase separation under physiological pH, ionic strength and haemoglobin concentrations^53^. These observations suggest that respiratory protein condensates may function as a universally conserved respiratory hub for tunable gas exchange. Although our findings provide specific evidence for the presence of PPO2 protein in in cellulo crystals, additional factors or phase-transition steps, ranging from liquid-to-gel or gel-to-solid states, may be involved in their formation. Moreover, the mechanisms underlying the acquisition of metastability in the solid-like state within a short time in vivo remain unclear. Future biochemical and biophysical studies of PPO2 crystals, complementing our genetic and cellular analyses, will shed light on the mechanisms of the PPO2 phase separation and the kinetics of crystal assembly in vivo.

Our study highlights that larval habitat, whether plastic vials in laboratory conditions or rotten fruits in the wild, inherently exposes *Drosophila* larvae to hypoxia. This environmental challenge forces the larvae to find a balance between fitness and immunity. While digging deeper provides better protection from predators, it potentially exposes them to hypoxia. Among the two major immune cell types in *Drosophila*, crystal cells have been recognized for their distinctive function in wound healing and melanization^36,39^. However, our study suggests that crystal cells offer a unique dual-protection mechanism through both innate immune responses and respiratory support, providing insights into the evolutionary origins of respiratory immune cells in animals. From a haematopoiesis perspective, crystal cells exhibit convergent functions resembling both platelets and erythrocytes, which are functionally analogous to megakaryocyte–erythrocyte lineage myeloid cells^35,54^. Furthermore, in other insects, haemocytes are often observed surrounding tracheal branches^55,56^, and other insects possess crystal cell equivalents known as oenocytoids^57,58^. These observations suggest that the association between haemocytes and the tracheal system for respiration and oxygen responsiveness may be conserved across insects and not limited to *Drosophila*.

## Methods

### *Drosophila* stocks and genetics

The following *Drosophila* stocks were used in this study: *Hml*^*Δ*^*-Gal4* (S. Sinenko), *Hml*^*Δ*^*-Gal4 UAS-2xeGFP* (S. Sinenko), *lz-LexA LexAop-mCherry* (J. Shim), *UAS-hid,rpr* (J. R. Nambu), *21-7-Gal4* (Y. N. Jan), *btl-Gal4* *UAS-GFP* (BL8807), *btl-Gal4* (BL78328)*, Hml*^*Δ*^*-dsRed* (K. Brueckner), *UAS-Notch*^*ICD*^ (U. Banerjee), *20xUAS-shi*^*ts*^*/TM6B* (A. J. Kim), *UAS-Gtpx* (W.-J. Lee), *tub-cyto-roGFP2-Orp1* (BL67670), *UAS-PPO2-V5* (W.-J. Lee), *tub-Gal80*^*ts*^ (BL7016), *btl* RNAi (BL43544), *lz*^*r15*^ (BL33835), *lz-Gal4 UAS-GFP* (BL6314), *20xUAS-6xmCherry-HA* (BL52268), *13xLexAop-6xmCherry-HA* (BL52271), *PPO2*^*Δ*^ (BL56205), *PPO1*^*Δ*^ (BL56204), *OK72-Gal4* (DGRC108801), *UAS-mCD8::GFP* (BL5137), *eater*^*1*^ (BL68388), *hs-Gal4 UAS-nlsTimer* (BL78057), *Notch-Gal4* (BL49528), *PPO2* RNAi (VDRC107772), *CAH2* RNAi (VDRC108184), *MtnA* RNAi (VDRC105011), *Atox1* RNAi (VDRC104437), *Ctr1A* RNAi (BL58107), *Punt* RNAi (VDRC37279), *Baboon* RNAi (VDRC3825), *dSmad2* RNAi (VDRC14609), *polo* RNAi (BL36093, BL33042, BL35146, BL36702), *stg* RNAi (BL34831, BL29556, BL36094), *ush* RNAi (BL32950, BL44041, BL29516), *Ras85D* RNAi (BL34619), *PPO1* RNAi (VDRC 107599), *fok* RNAi (BL63980), *CG10467* RNAi (BL62208), *Men* RNAi (BL38256), *CG15343* RNAi (VDRC101184), *Pde1c* RNAi (VDRC101906), *CG9119* RNAi (VDRC46326), *CG7860* RNAi (VDRC108281), *CG10469* RNAi (BL55291), *mthl10* RNAi (BL51753), *Gip* RNAi (VDRC105750), *CG17109* RNAi (BL54033), *Naxd* RNAi (VDRC39667), *peb* RNAi (BL28735), *tna* RNAi (BL29372), *Duox* RNAi (U. Banerjee) and *UAS-Sod2* (BL24494). *w*^*1118*^ (BL3605) and *Oregon R* (BL5) were used as wild types. *Hml*^*LT*^*-Gal4* is a lineage tracing of *Hml*^+^ haemocytes containing *Hml*^*Δ*^*-Gal4;UAS-FLP;ubi-FRT-STOP-FRT-Gal4*.

The following recombinants or combinations were generated in this study: *Hml*^*Δ*^*-Gal4* *UAS-eGFP;lz-LexA LexAop-mCherry*, *btl-Gal4* *UAS-GFP;lz-LexA* *LexAop-mCherry*, *UAS-mCD8::GFP;21-7-Gal4;Hml*^*Δ*^*-LexA* *LexAop-mCherry*, *UAS-mCD8::GFP;21-7-Gal4*, *lz-LexA* *LexAop-mCherry*, *lz*^*r15*^*;Hml*^*Δ*^*-Gal4 UAS-eGFP*, *ok72-Gal4* *UAS-mCD8::GFP; lz-LexA* *LexAop-mCherry*, *Hml*^*Δ*^*-dsRed;btl-Gal4 UAS-GFP*, *btl-Gal4* *UAS-GFP;lz-LexA* *LexAop-mCherry*, *21-7-Gal4* *UAS-mCD8::GFP;lz-LexA* *LexAop-mCherry*, *tub-Gal80*^*ts*^*;btl-Gal4* *UAS-GFP*, *PPO2*^*Δ*^*;hs-Gal4* *UAS-Timer, PPO2*^*Δ*^*;Notch-Gal4, PPO2*^*Δ*^*;UAS-PPO2*, *PPO2*^*Δ*^*;UAS-PPO2*^*H369N*^*, PPO2*^*Δ*^*;UAS-Timer* and *PPO2*^*Δ*^*;UAS-L.* *pol* *Hc2-Flag*.

The following stocks were generated in this study: *Hml*^*Δ*^*-LexA*, *lz-Gal4*, *UAS-PPO2*, *UAS-PPO2-eGFP*, *UAS-PPO2-Flag*, *UAS-PPO2*^*H369N*^*, UAS-PPO2*^*H212NH/369N*^*, UAS-PPO2*^*R50A*^*, UAS-PPO2*^*H212NH/369N*^*-Flag* and *UAS-L.pol* *Hc2-Flag*. For *Hml*^*Δ*^*-LexA*, the *Hml* enhancer was amplified from genomic DNA and cloned into a TOPO-TA vector (K252020; Thermo Fisher Scientific) for gateway cloning. The cloned entry vector was ligated into the pBPnlsLexA::p65Uw (26230; Addgene) destination vector using LR ligase (11791-020; Thermo Fisher Scientific). *lz-Gal4* was generated by splitting and rebalancing *lz-Gal4* *UAS-eGFP*, *20xUAS-6xmCherry-HA* with *Basc/FM7i;MKRS/TM6B*. For *UAS-PPO2*, *PPO2* cDNA was amplified from RNA extracted from larval haemocytes and cloned into the pGEM-T Easy vector (A1360; Promega). *PPO2* cDNA with restriction enzyme sites was amplified for ligation into the pUASTattB vector (1419; DGRC). For *UAS-PPO2-eGFP*, *PPO2* or *eGFP*, respectively, cDNA was amplified for ligation using a Gibson Assembly kit (E2611L; New England Biolabs). A linker (5′-GGCGGCGGCGGC-3′) was inserted between *PPO2* and *eGFP*. *PPO2-linker-eGFP* with a restriction enzyme site was amplified and ligated into the pUAST-attB vector (1419; DGRC). Mutagenesis of *UAS-PPO2* to produce *UAS-PPO2*^*H369N*^, *UAS-PPO2*^*R50A*^ and *UAS-PPO2*^*H369N/H212N*^ was performed using a mutagenesis kit (EZ004S; Enzynomics). PCR for *UAS-PPO2*^*WT*^*-Flag* or *PPO2*^*H369N/H212N*^*-Flag* was performed based on *UAS-PPO2* or *UAS-PPO2*^*H369N/H212N*^, respectively, using Flag primers. For *UAS-L.pol haemocyanin 2-Flag, L. polyphemus* *haemocyanin 2* gene fragments were synthesized by IDT; it was then amplified by PCR and cloned into the pGEM-T Easy vector (A1360; Promega). *L. polyphemus* *Hc2* cDNA was amplified with *Flag* primers for ligation into the pUASTattB vector (1419; DGRC). Detailed genotypes, sample sizes and Gal4 drivers with corresponding target tissues are listed in Supplementary Table 5. Experiments were independently repeated at least three times. A list of the primers used for cloning is provided in Supplementary Table 6.

Transgenic flies were generated by BestGene or KDRC. Unless indicated, all fly crosses and larvae were maintained at 25 °C and in normal dextrose–cornmeal-based food.

*tub-GAL80*^*ts*^*;btl-GAL4 UAS-GFP* crossed with *UAS-btl RNAi* flies or *21-7-GAL4 UAS-mCD8::GFP* crossed with *UAS-shi*^*ts*^ flies were maintained at 18 °C for 5 days (until the early second instar) and then transferred to 29 °C to avoid the larval lethal phenotype. Flies or larvae were randomly selected to perform all the experiments. Blinding was not applicable due to the complex genetic background and environmental conditions used in this study. All the data were collected based on unbiased analyses. Males and females of the same age, respectively, were used for experiments using flies. Unless indicated, the third-instar larvae at 120 hours after egg laying were used for experiments using larvae.

### O_2_ control experiments

All of the experiments were conducted in an O_2_/CO_2_ control chamber (ProOx Model C21; BioSpherix). For anoxia, hypoxia and hyperoxia experiments, 0.1%, 5% and 60% O_2_ were used, respectively. All larvae were synchronized and raised in the chamber for the indicated periods. Larvae were bled and imaged at 120 h AEL at 25 °C. For example, for 4 h hypoxia, larvae were synchronized and raised in normoxia until 116 h AEL and then transferred to 5% O_2_ at 116 h AEL until 120 h AEL. After 4 h in hypoxia, larvae were bled at 120 h AEL.

### Cell transfection

*Drosophila* S2R^+^ cells were maintained at 25 °C in Schneider’s medium (21720-024; Thermo Fisher Scientific) with 10% FBS, 50 U penicillin and 50 µg streptomycin per ml. To express Flag-tagged proteins, *Drosophila* S2R^+^ cells were transfected using the Cellfection reagent (58760; Thermo Fisher Scientific). UAS vector (*pUASt-PPO2-Flag*, *pUASt-PPO2*^*H212N/H369N*^*-Flag*, and *UAS L. polyphemus haemocyanin 2-Flag*) were co-transfected with *pAC5C-Gal4*. After transfection, S2R^+^ cells were incubated for 72 h before cell collection. The S2R^+^ cell line was confirmed by the morphology and was only used for protein purification.

### Immunoprecipitation and absorbance spectrum measurement

Immunoprecipitation was performed to isolate the Flag-tagged proteins from transfected *Drosophila* S2R^+^ cells. Cells were lysed in IP buffer (50 mM Tris-HCl, 50 mM NaCl, 300 mM sucrose, 1% Triton X-100, 0.2 mM PMSF) containing protease inhibitor cocktail (P9599; Sigma-Aldrich) on ice for 5 min after vortexing. Cell lysates were cleared by centrifugation at 12,000*g* and 4 °C for 15 min to remove cellular debris. Supernatants were collected and incubated with Anti-DYKDDDDK G1 Affinity Resin (L00432-1; GenScript Biotech) for 1 h at 4 °C with gentle rotation. The beads were washed three times with wash buffer (50 mM Tris-HCl, 50 mM NaCl, 300 mM sucrose, 0.2% Triton X-100, 0.2 mM PMSF, including protease inhibitor cocktail) to remove non-specific binding and the Flag-tagged proteins were eluted using 3× Flag peptide (F4799; Sigma-Aldrich) elution solution (150 ng µl^−1^ of 3× Flag peptide in 100 mM pH 7.5 Tris, 150 mM NaCl, 10 µM CuSO_4_) for 30 min at 4 °C. The 3× Flag peptide stock solution was made by dissolving 3× Flag peptide in 0.5 M Tris HCl, pH 7.5 and 1 M NaCl at a final concentration of 25 µM µl^−1^. Eluted proteins were then subjected to spectrophotometry analysis. Absorption spectra were measured using a spectrophotometer (Cary 60 UV-Vis; Agilent Technologies) at wavelengths of 200–800 nm at 0.5 nm intervals.

### Haemocyte bleeding

To bleed out the entire haemocyte population, including circulating and sessile cells, larvae were vortexed as described previously^59^ and bled on a glass slide (61.100.17; Immuno-Cell). Circulating haemocytes were obtained without any disturbance. After bleeding the larvae, haemocytes were allowed to settle for 40 min at 4 °C. Sessile haemocytes were collected by vigorously pipetting larval carcasses with PBS as described previously^1^. Haemocytes were fixed with a 3.7% formaldehyde solution, washed three times with 0.4% PBS-T (Triton X-100) for 10 min and blocked in 10% normal goat serum solution for 30 min. Primary antibody was added, and the slides containing haemocytes were incubated at 4 °C overnight. Haemocytes were washed three times with 0.4% PBS-T (Triton X-100) for 10 min and secondary antibody was added. The slides were incubated at room temperature for 3 h. Haemocytes were again washed three times with 0.4% PBS-T (Triton X-100) for 10 min each with a final wash with PBS for 3 min. Haemocyte samples were mounted in Vectashield (Vector Laboratories) with DAPI and imaged using a Nikon C2 Si-plus confocal microscope (Nikon).

### Live imaging of haemocytes

To visualize crystal assembly and dissolution ex vivo, we vortexed larvae for 2 min with glass bleeds (Sigma-Aldrich, G9268) as described above^59^. Larvae expressing *PPO1-Gal4* *UAS-PPO2-eGFP* were bled in 20 µl of Schneider’s medium (21720-024; Thermo Fisher Scientific) onto a glass-bottomed confocal dish (100350; SPL Life Sciences) and allowed to settle for 10 min. Haemocytes were washed with 20 µl Schneider’s medium and imaged using the Zeiss LSM 900 confocal microscope (Zeiss) with an incubation system at 25 °C at the Biospecimen-Multiomics Digital Bioanalysis Core Facility of Hanyang University. Humidity was maintained by the incubator.

### Haemocyte reattachment assay

To measure the number of haemocytes that returned to the haematopoietic pocket over 30 min, synchronized larvae grown until 116 h AEL were collected and cultured in a hypoxic chamber for 3.5 h. Larvae were collected in a tube, vortexed for 2 min with glass beads (Sigma-Aldrich, G9268) as described previously^59^ and placed back into the hypoxic chamber for another 30 min. After the 30 min incubation, larvae were bled onto a slide (61.100.17; Immuno-Cell International), and sessile or circulating haemocytes were counted.

### Live imaging of whole larvae

At 120 h AEL, synchronized larvae (*Hml*^*Δ*^*-Gal4 UAS-EGFP; lz-LexA LexAop-mCherry*) were placed in a larva-holding cassette (custom made by 3D printing). To prevent larvae from moving, larvae were covered by a cover glass (Deckglaser, 22 × 50 mm) and sealed on both sides with tape. Live imaging was recorded for 1 h using a Nikon A1 confocal microscope (Nikon) with an installed 5% O_2_ hypoxia chamber.

### Immunohistochemistry

The following primary antibodies were used in this study: anti-Pxn (1:1,000, rabbit)^60^, anti-Hnt (1:10, mouse; DSHB), anti-lz (1:10, mouse; DSHB), anti-PPO2 (1:1,000, rabbit), anti-Sima (1:1,000, guinea pig)^61^, anti-PH3 (1:500, rabbit; 06-570, Merck Millipore), anti-Flag (1:1,000, mouse; Sigma-Aldrich, F1804) and anti-DCP1 (1:100, rabbit; 9578, Cell Signaling).

Cy3-conjugated, 647-conjugated and FITC-conjugated secondary antibodies (Jackson Laboratory) were used at dilutions of 1:250.

To generate antisera specific to the PPO2 protein, a 6×His-tag fusion protein containing the entire PPO2 protein was produced using *Escherichia coli* (*pET21a-PPO2*). The recombinant PPO2-6×His proteins were purified and injected into rabbits to generate polyclonal antibodies (GenScript).

### TEM analysis

Ten third instar larvae were bled, collected and washed in PBS and fixed in 3% glutaraldehyde in 0.1 M cacodylate buffer (pH 7.2) containing 0.1% CaCl_2_ for 3 h at room temperature. This process was repeated until the desired amount. Haemocytes were washed five times with 0.1 M cacodylate buffer at 4 °C and were post-fixed with 1% OsO_4_ in 0.1 M cacodylate buffer containing 0.1% CaCl_2_ for 2 h at 4 °C. These samples were embedded in Embed-812 (EMS). After polymerization of the resin at 60 °C for 36 h, serial sections were cut with a diamond knife on a ULTRACUT UC7 ultramicrotome (Leica) and mounted onto formvar-coated slot grids. The sections were stained with 4% uranyl acetate for 10 min and lead citrate for 7 min. TEM imaging was conducted on a Tecnai G2 Spirit Twin transmission electron microscope (Thermo Fisher Scientific).

### RT–qPCR

More than 50 wandering third instar larvae were bled for haemocyte RNA extraction, and cDNA was synthesized using a quantitative PCR with reverse transcription (RT–qPCR) kit (TOYOBO). RT–qPCR was performed using the SYBR Green Master Mix and the comparative *C*_t_ method using the Step One-Plus Real-Time PCR thermal cycler (Thermo Fisher Scientific). Gene expression was normalized to *Rp49* expression, and a list of the specific primers used for RT–qPCR is provided in Supplementary Table 6.

### Imaging of whole circulating or sessile haemocytes

Larvae were placed on a glass slide with 100% glycerol and heated for 30 s on a 70 °C heat block^27^. Larvae were gently overlain with a cover glass without sealing. Whole circulating or sessile haemocytes were scanned using the Nikon C2 Si-plus confocal microscope (Nikon) or the Zeiss Axiocam 503 (Zeiss) system. In all of the images shown, the anterior is left, and the dorsal side is up.

### pHrodo green AM intracellular pH indicator

All of the steps were performed as described in the previous study, except for a few modifications^62^. Two larvae were bled in pHrodo green dye (P35373, Thermo Fisher Scientific) for 25 min at room temperature. Haemocytes were washed once with PBS for 4 min. Haemocyte samples were mounted in Vectashield (Vector Laboratories) and imaged immediately after mounting with a Nikon C2 Si-plus confocal microscope (Nikon). To create a standard curve using pHrodo green, two larvae were bled in pHrodo green dye and incubated for 25 min at room temperature. Haemocytes were washed once with Life Cell Imaging Solution (LCIS, A14291DJ, Thermo Fisher Scientific) for 3 min and then with buffer solutions of different pHs (pH 7.5, 6.5, 5.5 and 4.5) for 5 min each. Haemocyte samples were mounted in Vectashield (Vector Laboratories) with DAPI and imaged using a Nikon C2 Si-plus confocal microscope (Nikon). A linear function graph was constructed by obtaining GFP intensity values in each pH buffer. pH values were calculated from GFP intensities based on the graph. For image acquisition, three larvae were bled into one well, and the intensity was averaged by randomly selecting four sections. One dot indicates one trial, and each trial involves quantification of three larvae in one well.

### Tracheal TTBs

The procedure for TTB quantification was performed according to a previously described protocol^7^ with a few modifications. Wandering third instar larvae were mounted using the same procedure as for whole-larval imaging. TTBs of immobilized larvae were visualized under a bright-field microscope (Zeiss Axiocam 503, Zeiss). Dorsal views of segment T3 were magnified for TTB images, and TTBs on both sides were counted. Sample sizes (*n*) indicate the number of larvae counted. In all images, *z* stacks were analysed using ImageJ, and the anterior is up. Synchronized larvae were grown under normoxic conditions (21% O_2_) or shifted to either hypoxia (5% O_2_) or hyperoxia (60% O_2_) at 96 h AEL (hypoxia) or 60 h AEL (hyperoxia) until 120 h AEL. Excel v.16.58 (Microsoft) and Prism 9 (GraphPad) were used to calculate *P* values and to produce the final graphs.

### nlsTimer in *Drosophila* organs and haemocytes

For measuring nlsTimer in organs, all of the steps were performed as described in the previous study, except for a few modifications^47^. Synchronized first-instar larvae at 24 h AEL were collected and raised at 25 °C until 60 h AEL. Larvae were heat-shocked at 37 °C for 20 min followed by recovery at 18 °C for 6 h. After recovery, larvae were raised at 25 °C until 120 h AEL. For hypoxic conditions, larvae were raised in the 5% O_2_ chamber immediately after the 18 °C recoveries. At 120 h AEL, the brain, trachea, muscle, foregut, midgut, hindgut, salivary gland, fat body, eye disc and leg disc were collected in ice-cooled PBS. After 30 min of fixation with a 3.7% formaldehyde solution at 25 °C, the samples were washed one time each briefly in 0.4% PBS-T and then 1× PBS. After the final wash, the samples were maintained in Vectashield (Vector Laboratories) without DAPI.

For measuring nlsTimer in haemocytes, synchronized first-instar larvae at 24 h AEL were collected and raised at 25 °C until 120 h AEL. For hypoxic conditions, larvae were switched to the 5% O_2_ chamber and raised there from 60 h AEL to 120-h AEL. Larvae were bled at 120 h AEL. All of the samples were scanned with the Nikon C2 Si-plus confocal microscope (Nikon). Scan settings were described previously^47^.

### Larval survival rate

Forty synchronized larvae were transferred to individual vials and grown under 21% O_2_. After rearing until 120 h AEL, live larvae (>2.8 mm in length for third-instar larvae)^26^ were counted per vial. For survival rates in hypoxia or hyperoxia, synchronized larvae were transferred to individual vials at 24 h AEL and shifted to 5% O_2_ or 60% O_2_ until 120 h AEL. For survival rates in shallow food, synchronized larvae were cultured in normoxia and transferred to 3-mm-deep food at 24 h AEL. One dot represents one trial (*n* = 40). Excel v.16.58 (Microsoft) and Prism 9 (GraphPad) were used to calculate *P* values and produce the final graphs.

### Pupal volume

Synchronized larvae were cultured in normoxia and transferred to hypoxia or hyperoxia at 114 h AEL, and phenotypes were observed after pupariation at 144 h AEL. Pupal volume was measured using ImageJ (NIH) and calculated using the formula 4/3π(*L*/2)(*l*/2)^2^, where *L* is the length and *l* is the diameter^63^. Excel v.16.58 (Microsoft) and Prism 9 (GraphPad) were used to calculate *P* values and produce the final graphs.

### Quantification of samples, statistics and reproducibility

All haemocyte samples were visualized using the Zeiss Axiocam 503 (Zeiss) (2.5×) system with DAPI, GFP and RFP. ImageJ (NIH) was used to quantify circulating, sessile or total haemocytes. Imaris (Bitplane) was used to analyse crystal cell numbers in the lymph gland. For proximity measurements, Imaris (Bitplane) was used to calculate the proximity index. Each haemocyte (crystal cell, plasmatocyte) was made into a circle spot (threshold = 8 μm), and oenocytes, tracheal branching or neurons were made into 3D surfaces. Spots close to the surface were calculated (threshold = 5 μm) and normalized with their length.

For analysing haemocyte *nlsTimer* expression, all haemocyte samples were visualized using the Nikon C2 Si-plus confocal microscope (Nikon) (40×) with GFP and RFP. Two to four images were taken in each well (trial). Each haemocyte intensity was measured using ImageJ (NIH), and the M/G ratio was calculated. Other organs expressing *nlsTimer* were visualized using the Nikon C2 Si-plus confocal microscope (Nikon) (20×) with GFP and RFP. Twenty to thirty nuclei were measured per organ using ImageJ (NIH), and the M/G ratio was calculated. For analysing the ratio of crystalline to cytosolic structures among all PPO2^+^ crystal cells, one larva was placed on the bleeding slide at a time, and all PPO2^+^ cells were observed at 600× magnification through the Nikon C2 Si-plus confocal microscope (Nikon).

The detailed sample sizes for all experiments are indicated in Supplementary Table 5. At least three biologically independent samples were examined to perform statistical analyses. The centre values in all of the box and whisker plots in Figs. 1, 2, 4 and 5 and Extended Data Figs. 1–4 and 6–8 indicate the median values. The experiments shown in Fig. 3b were repeated four times, those in Figs. 3h and 4b were repeated three times and those in Extended Data Figs. 1f,h and 3i were repeated twice.

### BioTracker green copper live-cell dye

All of the steps were performed as described in a previous study^64^, except for a few modifications. Larvae were bled in BioTracker Green Copper dye (SCT041; Sigma-Aldrich) for 40 min at room temperature. Haemocytes were washed once with PBS for 4 min. Haemocyte samples were mounted in Vectashield (Vector Laboratories) and imaged immediately after mounting with the Nikon C2 Si-plus confocal microscope (Nikon).

### Shallow food preparation

A cornmeal, dextrose and yeast food recipe (Bloomington Drosophila Stock Center) was used in equivalent amounts but at different heights. Food was placed in a 60 mm × 15 mm Petri dish (10060; SPL Life Sciences) to a height of 3 mm, which was lower than the food usually provided for third-instar larvae.

### SABER-FISH

All of the steps were performed as described in a previous study except for a few modifications^65^. Three larvae were bled in 4% paraformaldehyde for 30 min. The samples were washed three times with 0.3% PBS-Tw (10× PBS + Tween-20 + H_2_O) for 5 min. Then, 0.3% PBS-Tw was replaced with wHyb (20× SSC + Tween-20 + formamide + H_2_O) and washed three times for 5 min. After the wHyb wash, the samples were replaced with Hyb1 (2× SSC + Tween-20 + formamide + 10% dextran sulfate) with probe mixture prewarmed at 43 °C and incubated for 32 h at 43 °C. After incubation, the samples were washed twice with wHyb for 30 min at 43 °C and replaced with 2× SSCT (2× SSC + Tween-20 + H_2_O) twice for 5 min at 43 °C, then 2× SSCT was replaced with 0.3% PBS-Tw twice for 5 min at 37 °C, and 0.3 PBS-Tw was replaced with wHyb twice for 5 min at 37 °C. The samples were replaced with wHyb to Hyb2/Flour solution (Flour Oligo Cy3 sequencer + H_2_O + Hyb2 solution (PBS + Tween-20 + dextran sulfate) prewarmed at 37 °C and incubated for 5 h at 37 °C and then replaced with 0.3% PBS-Tw twice for 10 min at 37 °C to wash. The samples were mounted in the Vectashield with DAPI (Vector Laboratory) and imaged using the Nikon C2 Si-plus confocal microscope (Nikon).

CAH2 probe sequences were as follows: (1) CGGGGTGTTGCGACACCTCCTC and (2) CACAAATCAAGATCGGAGCAATGACAATTG. The Flour Oligo Cy3 Imager sequence was as follows: TTATGATGATGTATGATGATGT.

### Reporting summary

Further information on research design is available in the Nature Portfolio Reporting Summary linked to this article.

## Online content

Any methods, additional references, Nature Portfolio reporting summaries, source data, extended data, supplementary information, acknowledgements, peer review information; details of author contributions and competing interests; and statements of data and code availability are available at 10.1038/s41586-024-07583-x.

## Supplementary information

Supplementary Tables 1–4

Reporting Summary

Supplementary Table 5 List of genetic backgrounds used in this study and corresponding sample sizes. List of detailed genotypes, statistical details and sample sizes used in the main figures or extended data figures. The first sheet indicates detailed genotypes and corresponding sample sizes for the main figures. The second sheet covers the Extended Data figures. The third and fourth sheets indicate statistical details of the main text and Extended Data figures, respectively. The fifth sheet describes the representative *Gal4* lines used in this study and their target tissues.

Supplementary Table 6 List of primers used in this study and detailed sequences. The first sheet indicates primers that were used in cloning and their sequence information. The second sheet indicates primers that were used in RT–qPCR.

Supplementary Video 1 3D rendering of PPO2 cubic crystalline form in the crystal cell.

Supplementary Video 2 3D rendering of PPO2 cylindrical crystalline form in the crystal cell.

Supplementary Video 3 3D rendering of PPO2 cytosolic form in the crystal cell.

Supplementary Video 4 Live imaging of dynamic changes during the PPO2 phase transition. Live imaging of in cellulo PPO2 crystal assembly and disassembly using *PPO1-Gal4;UAS-PPO2-Egfp*. GFP was imaged using DIC (Methods).

## Source data

Source Data Fig. 1–5 and Source Data Extended Data Fig. 1–8

## Data Availability

All data supporting the findings of this study are available in the Article and its Supplementary Information. We used Flybase release FB2024_02 to obtain the sequence, data and analysis in this study. Resources and reagents used in the study are available from the corresponding author on request. Source data are provided with this paper.
