# Author Correction: *Drosophila* immune cells transport oxygen through PPO2 protein phase transition

**DOI:** 10.1038/s41586-024-08058-9

**Published:** 2024-09-20

**Authors:** Mingyu Shin, Eunji Chang, Daewon Lee, Nayun Kim, Bumsik Cho, Nuri Cha, Ferdinand Koranteng, Ji-Joon Song, Jiwon Shim

**Affiliations:** 1 Department of Life Science, College of Natural Science, Hanyang University, Seoul, Republic of Korea; 2 Department of Biological Sciences, KI for BioCentury, Korea Advanced Institute of Science and Technology (KAIST), Daejeon, Republic of Korea; 3 Research Institute for Natural Science, Hanyang University, Seoul, Republic of Korea; 4 Hanyang Institute of Bioscience and Biotechnology, Hanyang University, Seoul, Republic of Korea; 5 Hanyang Institute of Advanced BioConvergence, Hanyang University, Seoul, Republic of Korea

**Keywords:** Respiration, Haematopoiesis, Cellular imaging, Evolutionary developmental biology

Correction to: *Nature* 10.1038/s41586-024-07583-x Published online 26 June 2024

The authors have been informed that in the version of this article initially published, the term “haem” was used incorrectly in connection with the pigment haemocyanin. Text has been clarified in the third paragraph of the “O_2_ establishes PPO2 in cellulo crystals” section, in the second paragraph of the “Phase transition of PPO2 and oxygenation” section and in the Extended Data Fig. 5c legend. The changes are made in the HTML and PDF versions of the article.

