# Supplementary material for: Drosophila immune cells transport oxygen through PPO2 protein phase transition
Source: Nature. 2024 Jun 26;631(8020):350–9. doi: 10.1038/s41586-024-07583-x (PMC11236712; doi:10.1038/s41586-024-07583-x)
Supplement: Supplementary file 1 — Supplementary Tables 1–4 [file 41586_2024_7583_MOESM1_ESM.pdf]

nature portfolio

https://doi.org/10.1038/s41586-024-07583-x

Supplementary information

Drosophila immune cells transport oxygen through PPO2 protein phase transition

In the format provided by the authors and unedited

Supplementary Information Guide

Drosophila immune cells transport oxygen via PPO2 protein phase transition

Mingyu Shin[1†], Eunji Chang[1†], Daewon Lee[1†], Nayun Kim[2], Bumsik Cho[1], Nuri Cha[1], Ferdinand Koranteng[1], Ji-Joon Song[2], and Jiwon Shim[1,3,4,5*]

[1]Department of Life Sciences, College of Natural Science, Hanyang University, Seoul, Republic of Korea 04763

[2]Department of Biological Sciences, KI for BioCentury, Korea Advanced Institute of Science and Technology (KAIST), Daejeon, Republic of Korea 34141

[3]Research Institute for Natural Science, Hanyang University, Seoul, Republic of Korea 04763

[4]Hanyang Institute of Bioscience and Biotechnology, Hanyang University, Seoul, Republic of Korea 04763

[5]Research Institute for Convergence of Basic Science, Hanyang University, Seoul, Republic of Korea 04763

[†]These authors contributed equally to this work.

[*]Corresponding author: jshim@hanyang.ac.kr

Supplementary Table 1. Numbers of thick terminal branches (TTBs) in different genetic backgrounds and oxygen conditions.

Thick terminal branches in the trachea were counted in different genetic backgrounds and oxygen concentrations. The first column indicates genotype and oxygen concentrations (21% O_2, normoxia; 5%, hypoxia; 60%, hyperoxia). The second column indicates the mean value of tracheal thick terminal branching with standard errors (S.E.). The third column indicates the number of larvae (n). The fourth column indicates the variation values compared to 100% in controls. The fifth column indicates the corresponding exact p value to each experiment and the sixth column indicates the p value summary. All data in the table were analyzed by the Mann–Whitney test.

Supplementary Table 2. Numbers of thick terminal branches (TTBs) with 95% confidence interval

The number of thick terminal branches (TTBs) in Supplementary Figure 1 was analyzed with a 95% confidence interval. Gray shades indicate groups of genotypes or conditions. The orange shading indicates the 95% confidence interval.

Supplementary Table 3. List of 20 most highly expressed genes in crystal cells.

The top 20 crystal-cell-enriched genes were extracted from single-cell RNA sequencing. The first column shows the abbreviation of the gene name in Flybase and the full name in parentheses. The second column shows the information on the annotation symbol of each gene based on FlyBase. The third column shows the FlyBase IDs of each gene.

Supplementary Table 4. List of crystal-cell-specific genes regulating hemocyte location.

List of five genes that control the hemocyte movement and the protein phase of PPO2. The first column shows the gene names and the second column shows the information on the annotation symbol and FlyBase ID of each gene. The third column describes the function of each gene.

Supplementary Table 1

Genotype, [O_2]	Thick Terminal	N	Variation	p value	summary
w^{1118}, 21% O_2	5.94±0.11	102	100%		
w^{1118}, 5% O_2	8.08±0.09^A	124	136%	<0.0001	****
w^{1118}, 60% O_2	5.32±0.10^A	103	89%	<0.0001	****
w^{1118}, 21% O_2 (3mm food)	5.67±0.13^A	39	95%	0.1476	ns
lz^{r15}, 21% O_2	8.33±0.13^A	92	140%	<0.0001	****
lz^{r15}, 5% O_2	8.47±0.11^B	102	142%	0.7586	ns
lz^{r15}, 60% O_2	6.74±0.11^B	85	113%	<0.0001	****
lz^{r15}, 21% O_2 (3mm food)	7.12±0.14^B	39	116%	0.0004	***
$PPO2^{\Delta}$, 21% O_2	8.27±0.13^A	104	139%	<0.0001	****
$PPO2^{\Delta}$, 5% O_2	8.49±0.12^C	102	143%	0.7408	ns
$PPO2^{\Delta}$, 60% O_2	6.68±0.10^C	98	113%	<0.0001	****
$PPO2^{\Delta}$, 21% O_2 (3mm food)	7.30±0.14^C	57	123%	<0.0001	****
$PPO2^{\Delta}$; Notch>PPO2, 21% O_2	7.40±0.10^C	99	90%	<0.0001	****
$PPO2^{\Delta}$; Notch>$PPO2^{H369N}$, 21% O_2	8.32±0.12^F	74	101%	<0.0001	****
$PPO2^{\Delta}$; Notch>L.pol Hc2, 21% O_2	7.05±0.11^C	32	117%	<0.0001	****
$PPO1^{\Delta}$, 21% O_2	6.91±0.12^A	107	116%	0.0002	***
$PPO1^{\Delta}$, 5% O_2	8.10±0.12^H	72	136%	0.001	**
$PPO1^{\Delta}$, 60% O_2	6.28±0.15^H	46	106%	0.0849	ns
$eater^{1}$, 21% O_2	8.07±0.17^A	91	128%	<0.0001	****
lz>GFP/+, 21% O_2	6.5±0.10	106	100%		
lz>GFP/+, 5% O_2	8.16±0.11^D	100	125%	<0.0001	****
lz>GFP/+, 60% O_2	6.01±0.09^D	81	92%	0.0028	**
lz>GFP/PPO2 RNAi, 21% O_2	8.46±0.12^D	107	130%	<0.0001	****
lz>GFP/PPO2 RNAi, 5% O_2	8.34±0.08^E	114	128%	0.4333	ns
lz>GFP/PPO2 RNAi, 60% O_2	6.58±0.13^E	60	101%	<0.0001	****
Hml>GFP/+, 21% O_2	6.33±0.11	90	100%		
Hml>GFP/PPO2 RNAi, 21% O_2	6.19±0.10^G	98	98%	0.2974	ns
Hml>GFP/Notch RNAi, 21% O_2	7.71±0.12^G	69	124%	<0.0001	****
Hml>GFP/ush RNAi, 21% O_2	6.11±0.19^G	27	98%	0.2702	ns
Hml>GFP/stg RNAi, 21% O_2	6.4±0.2^G	20	103%	0.6225	ns

Values are means ± SE.
^{A} Significant differences compared to w^{1118} control larvae.
^{B} Significant differences compared to lz^{r15} larvae.
^{C} Significant differences compared to $PPO2^{\Delta}$ larvae.
^{D} Significant differences compared to lz>GFP/+ control larvae.
^{E} Significant differences compared to lz>GFP/PPO2 RNAi larvae.
^{F} Significant differences compared to $PPO2^{\Delta}$; Notch>PPO2 larvae.
^{G} Significant differences compared to Hml>GFP larvae.
^{H} Significant differences compared to $PPO1^{\Delta}$ larvae.
(p<0.001: Mann Whitney test)

Supplementary Table 2

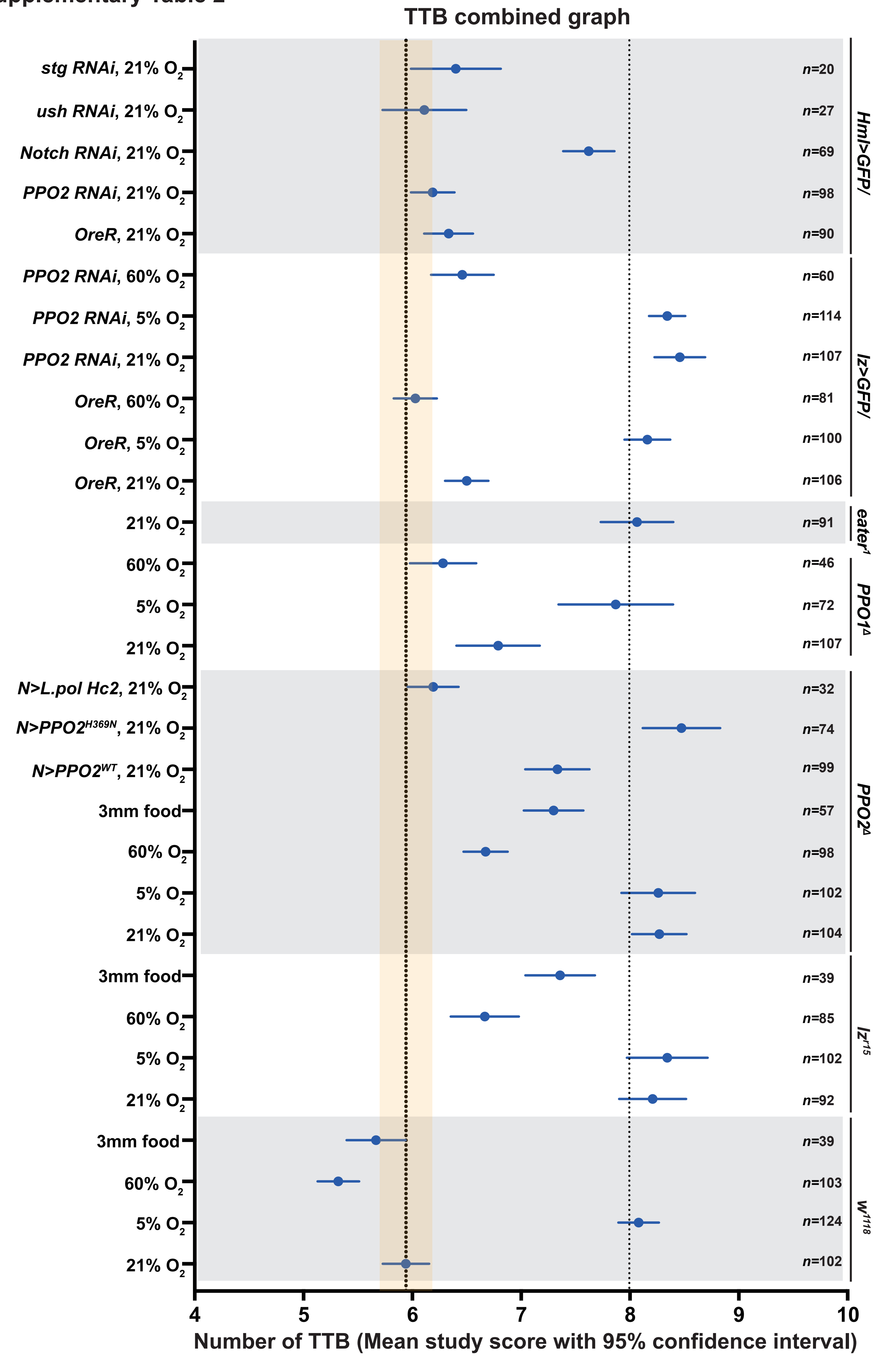

Supplementary Table 3

List of Top20 genes in Crystal cell

Gene	Annotation symbol	FlyBase ID
PPO2<br>(Prophenol Oxidase 2)	CG8193	FBgn0033367
PPO1<br>(Prophenol Oxidase 1)	CG42639	FBgn0283437
MtnA<br>(Metallothionein A)	CG9470	FBgn0002868
fok<br>(fledgling of Klp38B)	CG43690	FBgn0263773
CG10467<br>(Predicted to enable<br>aldose 1-epimerase activity)	CG10467	FBgn0035679
Men<br>(Malic enzyme)	CG10120	FBgn0002719
CG15343<br>(Predicted to enable<br>pyridoxamine-phosphate oxidase activity)	CG15343	FBgn0030029
Pde1c<br>(Phosphodiesterase 1c)	CG44007	FBgn0264815
CG9119<br>(Predicted to enable hydrolase activity)	CG9119	FBgn0035189
CG7860<br>(L-asparaginase<br>and beta-aspartyl peptidase activity)	CG7860	FBgn0030653
CG10469<br>(Enables serine hydrolase activity)	CG10469	FBgn0035678
Atox1<br>(Antioxidant 1 copper chaperone)	CG32446	FBgn0052446
mthl10<br>(methuselah-like 10)	CG17061	FBgn0035132
Gip<br>(GIP-like)	CG2227	FBgn0011770
CG17109<br>(Predicted to enable<br>aminoacylase activity.)	CG17109	FBgn0039051
Ctr1A<br>(Copper transporter 1A)	CG3977	FBgn0062413
Naxd<br>(NAD(P)HX dehydratase)	CG10424	FBgn0036848
peb<br>(pebbled)	CG12212	FBgn0003053
tna<br>(tonalli)	CG7958	FBgn0026160
CAH2<br>(Carbonic anhydrase)	CG6906	FBgn0027843

Supplementary Table 4

List of genes changing hemocyte location

Gene	Annotation symbol/FlyBase ID	Function
PPO2 (Prophenol Oxidase 2)	CG8193/ FBgn0033367	Involved in the melanization reaction
CAH2 (Carbonic anhydrase)	CG6906/ FBgn0027843	Hydration/dehydration of carbon dioxide to bicarbonate and protons
MtnA (Metallothionein A)	CG9470/ FBgn0002868	Role in the protection against metal toxicity and oxidative stress
Atox1 (Antioxidant 1 copper chaperone)	CG32446/ FBgn0052446	A metallochaperone that has metal trafficking and sequestration roles
Ctr1A (Copper transporter 1A)	CG3977/ FBgn0062413	Plasma membrane protein that functions as a copper transporter
