# Supplementary material for: Drosophila immune cells transport oxygen through PPO2 protein phase transition
Source: Nature. 2024 Jun 26;631(8020):350–9. doi: 10.1038/s41586-024-07583-x (PMC11236712; doi:10.1038/s41586-024-07583-x)
Supplement: Supplementary file 2 — Reporting Summary [file 41586_2024_7583_MOESM2_ESM.pdf]

nature portfolio

Corresponding author(s): Jiwon Shim

Last updated by author(s): May 5, 2024

Reporting Summary

Nature Portfolio wishes to improve the reproducibility of the work that we publish. This form provides structure for consistency and transparency in reporting. For further information on Nature Portfolio policies, see our Editorial Policies and the Editorial Policy Checklist.

Please do not complete any field with "not applicable" or n/a. Refer to the help text for what text to use if an item is not relevant to your study.
For final submission: please carefully check your responses for accuracy; you will not be able to make changes later.

Statistics

For all statistical analyses, confirm that the following items are present in the figure legend, table legend, main text, or Methods section.

n/a	Confirmed	
☐	☒	The exact sample size (*n*) for each experimental group/condition, given as a discrete number and unit of measurement
☐	☒	A statement on whether measurements were taken from distinct samples or whether the same sample was measured repeatedly
☐	☒	The statistical test(s) used AND whether they are one- or two-sided<br>*Only common tests should be described solely by name; describe more complex techniques in the Methods section.*
☒	☐	A description of all covariates tested
☐	☒	A description of any assumptions or corrections, such as tests of normality and adjustment for multiple comparisons
☐	☒	A full description of the statistical parameters including central tendency (e.g. means) or other basic estimates (e.g. regression coefficient) AND variation (e.g. standard deviation) or associated estimates of uncertainty (e.g. confidence intervals)
☐	☒	For null hypothesis testing, the test statistic (e.g. *F*, *t*, *r*) with confidence intervals, effect sizes, degrees of freedom and *P* value noted<br>*Give P values as exact values whenever suitable.*
☒	☐	For Bayesian analysis, information on the choice of priors and Markov chain Monte Carlo settings
☐	☒	For hierarchical and complex designs, identification of the appropriate level for tests and full reporting of outcomes
☒	☐	Estimates of effect sizes (e.g. Cohen's *d*, Pearson's *r*), indicating how they were calculated

Our web collection on statistics for biologists contains articles on many of the points above.

Software and code

Policy information about availability of computer code

Data collection	Confocal microscopy images were acquired using a Ziess LSM900 Airyscan2 or a Nikon C2 Si-Plus. All hemocyte samples or tracheal terminal branchings were visualized by a Zeiss Axiocam 503. Transmission electron microscopy imaging was conducted on a Tecnai G2 Spirit Twin transmission electron microscope (Thermo Fisher Scientific, USA).
Data analysis	Immunostaining and hemocyte samples were analyzed using Fiji 2.14.0 (General Public license, https://imagej.net/Fiji) or Imaris 8.4.1 (Bitplane, Belfast,UK). Statistical analyses and data blottings were performed using Prism 9.4.1 (GraphPad, La Jolla, CA, USA) or Excel version 16.58 (Microsoft Corp, Redmond, WA, USA).

For manuscripts utilizing custom algorithms or software that are central to the research but not yet described in published literature, software must be made available to editors and reviewers. We strongly encourage code deposition in a community repository (e.g. GitHub). See the Nature Portfolio guidelines for submitting code & software for further information.

Data

Policy information about availability of data

All manuscripts must include a data availability statement. This statement should provide the following information, where applicable:

- Accession codes, unique identifiers, or web links for publicly available datasets
- A description of any restrictions on data availability
- For clinical datasets or third party data, please ensure that the statement adheres to our policy

All data supporting the findings of this study are available in the main text or the supplementary materials. We used Flybase release (FB2024_02) to obtain the sequence/data/analysis resources used in this study. Resources and reagents used in the study are available upon request.

Research involving human participants, their data, or biological material

Policy information about studies with human participants or human data. See also policy information about sex, gender (identity/presentation), and sexual orientation and race, ethnicity and racism.

Reporting on sex and gender	human sources were not used in the experiment.
Reporting on race, ethnicity, or other socially relevant groupings	human sources were not used in the experiment.
Population characteristics	human sources were not used in the experiment.
Recruitment	human sources were not used in the experiment.
Ethics oversight	human sources were not used in the experiment.

Note that full information on the approval of the study protocol must also be provided in the manuscript.

Field-specific reporting

Please select the one below that is the best fit for your research. If you are not sure, read the appropriate sections before making your selection.

☒ Life sciences ☐ Behavioural & social sciences ☐ Ecological, evolutionary & environmental sciences

For a reference copy of the document with all sections, see nature.com/documents/nr-reporting-summary-flat.pdf

Life sciences study design

All studies must disclose on these points even when the disclosure is negative.

Sample size	The sample size was defined to stabilize relevant statistical parameters (median or mean). The sample size of all experiments was provided in Supplementary Table 5.
Data exclusions	No data was excluded from the analyses.
Replication	The experiments reported in this study were minimally performed in duplicates and most were triplicated (Biological repeats in two or more independent experimental settings). All attempts of replication were successful.
Randomization	Collected flies were always randomly selected to perform all the experiments.
Blinding	Blinding is not applicable due to complex genetic backgrounds and environmental conditions used in this study. Each experimental condition of samples must be known to perform experiments. However, all the data in this study were collected based on unbiased analysis.

Reporting for specific materials, systems and methods

We require information from authors about some types of materials, experimental systems and methods used in many studies. Here, indicate whether each material, system or method listed is relevant to your study. If you are not sure if a list item applies to your research, read the appropriate section before selecting a response.

Materials & experimental systems

n/a	Involved in the study
☐	☒ Antibodies
☐	☒ Eukaryotic cell lines
☒	☐ Palaeontology and archaeology
☐	☒ Animals and other organisms
☒	☐ Clinical data
☒	☐ Dual use research of concern
☒	☐ Plants

Methods

n/a	Involved in the study
☒	☐ ChIP-seq
☒	☐ Flow cytometry
☒	☐ MRI-based neuroimaging

Antibodies

Antibodies used

The following primary antibodies were used for immunostaining:
1) Commercial antibody
α-Hnt (1:10, mouse, 1G9, DSHB)
α-lz (1:10, mouse, anti-lozenge, DSHB)
α-PH3 (1:500, rabbit; #06-570, Merck Millipore)
α-FLAG (1:1000, mouse; Sigma F1804)
α-DCP1 (1:100, rabbit; #9578, Cell Signaling)
2) Shared antibody
α-Pxn (1:1000, rabbit, Yoon et al.,2017)
α-Sima (1:1000, guinea pig, Wang et al., 2016)
3) Antibody generated in this study
α-PPO2 (1:2000, rabbit, GenScript, Order number U606THE200)

The following secondary antibodies were used for immunostaining:
FITC-conjugated AffiniPure Goat anti-Mouse IgG (115-095-062)
Cy3-conjugated AffiniPure Goat anti-Mouse IgG (115-165-166)
Alexa Flour 647-conjugated AffiniPure Goat anti-Mouse IgG (115-605-003)
FITC-conjugated AffiniPure Donkey anti-Rabbit IgG (711-095-152)
Cy3-Conjugated AffiniPure Donkey anti-Rabbit IgG (711-165-152)
Alexa Flour 647-conjugated AffiniPure Donkey anti-Rabbit IgG (711-605-152)
FITC-conjugated AffiniPure Donkey anti-Guinea Pig IgG (706-095-148)
Cy3-conjugated AffiniPure Donkey anti-Guinea Pig IgG (706-165-148)
All the secondary antibodies above were provided from Jackson Laboratory, Bar Harbor, ME, USA and were used at dilutions of 1:250.

Validation

α-Hnt (1G9, DSHB) was validated for immunohistochemistry (PMID: 9187140), and deposited to the DSHB by Lipshitz, H.D.
α-lz (anti-lozenge, DSHB) was validated for immunohistochemistry (PMID: 10753120), and deposited to the DSHB by Banerjee, U.
α-PH3 (#06-570, Merck Millipore) was used for immunohistochemistry (PMID: 29622651). There are more references in the manufacturer's website.
α-FLAG (1:1000, mouse, F1804, Sigma) was validate for immunohistochemistry (PMID: 27630089). There are more references in the manufacturer's website.
α-DCP1 (1:100, rabbit; #9578, Cell Signaling) was validate for immunohistochemistry (PMID: 27058168). There are more references in the manufacturer's website.
α-Pxn was validated for immunohistochemistry and generated in PMID: 29237257.
α-Sima was validated for immunohistochemistry (PMID: 27585295).
α-PPO2 (rabbit, GenScript, U606THE200) was generated in this study and was validated for immunohistochemistry by PPO2-FLAG overexpression and PPO2 mutant background.

Eukaryotic cell lines

Policy information about cell lines and Sex and Gender in Research

Cell line source(s)

S2R+ cell lines
Species : Drosophila melanogatser
Source : Oregon R (Cherbas, 2008.11.12)
Tissue source : embryo (Schneider, 1972)
Developmental stage : late embryonic stage (Cherbas, 2008.11.12)
Lab or origin : Schneider (Schneider, 1972)
S2R+ (Stock number #150, DGRC) cell line was provided by the Drosophila Genomics Resource Center (DGRC) in Bloomington, IN, USA.

Authentication

Morphology of S2R+ cells were validated.

Mycoplasma contamination

All cell lines tested negative for mycoplasma contamination (DGRC).

Commonly misidentified lines
(See ICLAC register)

Misidentified cell lines were not used.

Animals and other research organisms

Policy information about studies involving animals; ARRIVE guidelines recommended for reporting animal research, and Sex and Gender in Research

Laboratory animals	One- or two-day old adult flies were used for mating. After egg collection, animals were raised in the Cornmeal/dextrose/yeast media until the desired timepoint. Unless specific developmental stage was indicated in Figure or Figure Legend, late third instar (120h after egg laying at 25'C) larva was used.<br><br>The following Drosophila stocks were used in this study.<br>Hml�-Gal4 (S. Sinenko), Hml�-Gal4 UAS-2xEGFP (S. Sinenko), lz-LexA LexAop-mCherry (J. Shim), UAS-hid, rpr (J.R. Nambu), 21-7-Gal4 (Y.N. Jan), btl-Gal4 UAS-GFP (BL8807), btl-Gal4 (BL78328), Hml�-dsRed (K. Brueckner), UAS-NotchICD (U. Banerjee), 20xUAS-shits/TM6B (A.J. Kim), UAS-Gtpx (W.J. Lee), tub-cyto-roGFP2-Orp1 (BL67670), UAS-PPO2-V5 (W.J. Lee), tub-Gal80ts (BL7016), btl RNAi (BL43544), lzr15 (BL33835), lz-Gal4 UAS-GFP (BL6134), 20xUAS-6xmCherry-HA (BL52268), 13xLexAop-6xmCherry-HA (BL52271), PPO2� (BL56205), PPO1� (BL56204), OK72-Gal4 (BL6486), UAS-mCD8::GFP (BL5137), eater1 (BL68388), hs-Gal4 UAS-nlsTimer (BL78057), Notch-Gal4 (BL49528), PPO2 RNAi (VDRC107772), CAH2 RNAi (VDRC108184), MtnA RNAi (VDRC105011), Atox1 RNAi (VDRC104437), Ctr1A RNAi (BL58107), Punt RNAi (VDRC37279), Baboon RNAi (VDRC3825), dSmad2 RNAi (VDRC14609), polo RNAi (BL36093, BL33042, BL35146, BL36702), stg RNAi (BL34831, BL29556, BL36094), ush RNAi (BL32950, BL44041, BL29516), Ras85D RNAi (BL34619), PPO1 RNAi (VDRC 107599), fok RNAi (BL63980), CG10467 RNAi (BL62208), Men RNAi (BL38256), CG15343 RNAi (VDRC101184), Pde1c RNAi (VDRC101906), CG9119 RNAi (VDRC46326), CG7860 RNAi (VDRC108281), CG10469 RNAi (BL55291), mthl10 RNAi (BL51753), Gip RNAi (VDRC105750), CG17109 RNAi (BL54033), Naxd RNAi (VDRC39667), peb RNAi (BL28735), tna RNAi (BL29372), Duox RNAi (U. Banerjee), and UAS-Sod2 (BL24494), w1118 (BL3605), Oregon R (BL5), Hml�-Gal4; UAS-FLP; ubi-FRT-STOP-FRT-Gal4, Hml�-Gal4 UAS-EGFP; lz-LexA LexAop-mCherry, btl-Gal4 UAS-GFP; lz-LexA LexAop-mCherry, UAS-mCD8::GFP; 21-7-Gal4; Hml�-LexA LexAop-mCherry, UAS-mCD8::GFP; 21-7-Gal4, lz-LexA LexAop-mCherry, lzr15; Hml�-Gal4 UAS-EGFP, ok72-Gal4 UAS-mCD8::GFP; lz-LexA LexAop-mCherry, Hml�-dsRed; btl-Gal4 UAS-GFP, btl-Gal4 UAS-GFP; lz-LexA LexAop-mCherry, 21-7-Gal4 UAS-mCD8::GFP; lz-LexA LexAop-mCherry, tub-Gal80ts; btl-Gal4 UAS-GFP, PPO2�; hs-Gal4 UAS-Timer, PPO2�; Notch-Gal4, PPO2�; UAS-PPO2, PPO2�; UAS-PPO2H369N, PPO2�; UAS-Timer, and PPO2�; UAS-L. pol Hc2-FLAG. Hml�-LexA, lz-Gal4, UAS-PPO2, UAS-PPO2-EGFP, UAS-PPO2-FLAG, UAS-PPO2H369N, UAS-PPO2H212NH/369N, UAS-PPO2R50A, UAS-PPO2H212NH/369N-FLAG, and UAS-L.pol Hc2-FLAG.
Wild animals	None
Reporting on sex	To measure the changes in pupal volume according to the oxygen concentration, the sex was divided and the experiment was conducted after the division according to the male/female classification method during the larval period.
Field-collected samples	None
Ethics oversight	None

Note that full information on the approval of the study protocol must also be provided in the manuscript.

This checklist template is licensed under a Creative Commons Attribution 4.0 International License, which permits use, sharing, adaptation, distribution and reproduction in any medium or format, as long as you give appropriate credit to the original author(s) and the source, provide a link to the Creative Commons license, and indicate if changes were made. The images or other third party material in this article are included in the article's Creative Commons license, unless indicated otherwise in a credit line to the material. If material is not included in the article's Creative Commons license and your intended use is not permitted by statutory regulation or exceeds the permitted use, you will need to obtain permission directly from the copyright holder. To view a copy of this license, visit http://creativecommons.org/licenses/by/4.0/
